# HNF4A-AS1 inhibits the progression of hepatocellular carcinoma by promoting the ubiquitin-modulated degradation of PCBP2 and suppressing the stability of ARG2 mRNA

**DOI:** 10.7150/ijbs.95276

**Published:** 2024-09-23

**Authors:** Wenbo Jia, Liang Yu, Bin Xu, Yanzhi Feng, Jinyi Wang, Deming Zhu, Chao Xu, Litao Liang, Yongping Zhou, Lianbao Kong, Wenzhou Ding

**Affiliations:** 1 Hepatobiliary Centre, The First Affiliated Hospital of Nanjing Medical University, Nanjing, Jiangsu Province, China.; 2 Key Laboratory of Liver Transplantation, Chinese Academy of Medical Sciences, National Health Commission (NHC) Key Laboratory of Living Donor Liver Transplantation (Nanjing Medical University), Nanjing, Jiangsu Province, China.; 3 Department of Hepatobiliary, Jiangnan University Medical Center, JUMC, China.; 4 Department of General Surgery, the Second Affiliated Hospital of Anhui Medical University, China.

**Keywords:** Hepatocellular carcinoma, HNF4A-AS1, PCBP2, ubiquitination, AGR2.

## Abstract

Hepatocellular carcinoma (HCC) is a highly aggressive malignant tumor with a poor prognosis. Extensive research has revealed the significant role of long noncoding RNAs (lncRNAs) in the regulation of tumor development. In this study, high-throughput sequencing analysis was used to assess the expression levels of lncRNAs in three pairs of HCC tissues and their corresponding noncancerous tissues. Through quantitative real-time polymerase chain reaction (qRT-PCR) analysis and clinicopathological analysis, it was discovered that HNF4A-AS1 was downregulated in HCC tissues. Furthermore, its expression levels were found to be positively correlated with the prognosis of HCC patients. Subsequent *in vitro* and *in vivo* functional studies demonstrated that HNF4A-AS1 inhibits the proliferation, invasion, and stemness of HCC cells. Mechanistically, it was observed that HNF4A-AS1 physically interacts with the KH3 domain of PCBP2 through a specific segment (491-672 nt). This interaction facilitates the recruitment of PCBP2 by AIP4, leading to the ubiquitination and subsequent degradation of PCBP2. Furthermore, HNF4A-AS1 was found to regulate the stability of AGR2 mRNA by modulating PCBP2, thereby influencing the malignant phenotype of HCC. Overall, our study demonstrated a positive association between the decrease in HNF4A-AS1 expression and the prognosis of patients with HCC in a clinical setting. HNF4A-AS1 can suppress the stability of ARG2 mRNA by promoting the ubiquitin-modulated degradation of PCBP2, which suppresses HCC progression. HNF4A-AS1 may serve as a potential therapeutic target for HCC.

## Introduction

Primary liver cancer is a prevalent malignant tumor that ranks sixth in terms of frequency and third in terms of cancer-related mortality worldwide^1^. HCC accounts for approximately 90% of all cases of primary liver cancer^2^, ^3^. Despite significant advancements in HCC treatment, the prognosis for patients remains suboptimal, primarily due to the high rates of recurrence and metastasis. Consequently, there is an urgent need to investigate the underlying pathological mechanisms of HCC in order to develop novel therapeutic approaches.

LncRNAs are a class of RNA molecules that exceed 200 nucleotides in length and possess limited or no protein-coding capacity^4^. These lncRNAs play diverse roles in fundamental biological processes, such as embryonic pluripotency, differentiation, and development, and they are closely associated with the initiation and progression of cancer^5^, ^6^. There is an increasing body of evidence indicating that lncRNAs have intricate and precise regulatory functions in the initiation and progression of cancer, serving as either oncogenes or tumor suppressors^7^, ^8^. These lncRNAs modulate the transcription and translation of genes associated with cancer through diverse mechanisms, such as acting as competing endogenous RNAs (ceRNAs) and scaffolds^9^, ^10^. Furthermore, lncRNAs influence the progression of cancer by affecting post-translational modifications of crucial cancer-related proteins, including ubiquitination, phosphorylation, and acetylation^11^-^13^. A subset of lncRNAs has been extensively studied in HCC. For example, USP27X-AS1 enhances USP7-mediated deubiquitination and activation of AKT to facilitate HCC progression^11^. Additionally, lncRNAs have been implicated in the development of drug resistance in HCC cells. Knockdown of Linc01056 contributes to sorafenib resistance by activating PPARα, thereby promoting fatty acid oxidation^14^. The significant roles and distinct properties of lncRNAs underscore their potential clinical significance in the diagnosis and treatment of HCC. However, the specific roles of most lncRNAs in HCC are not fully elucidated. Further comprehensive investigations are necessary to determine the precise mechanisms and functions of lncRNAs in HCC.

Poly(rC)-binding Protein 2 (PCBP2) is a member of the poly(C)-binding protein family and functions as an RNA-binding protein^15^. Its role in regulating tumor progression involves binding to poly(C), influencing intracellular transcriptional and post-transcriptional processes such as pre-mRNA splicing, mRNA stabilization, and translational control^16^, ^17^. PCBP2 plays a crucial role in modulating the malignant characteristics of HCC. For example, circCPSF promotes the proliferation and metastasis of HCC cells by interacting with PCBP2 and enhancing YAP1 expression^18^. Nevertheless, the precise mechanism underlying the interaction between PCBP2 and lncRNAs in the regulation of HCC progression remains to be elucidated.

The objective of this study was to investigate the regulatory function of Hepatocyte nuclear factor 4 alpha antisense RNA 1 (HNF4A-AS1) in HCC. Through RNA sequencing analysis and TCGA data analysis, we found that HNF4A-AS1 is downregulated in HCC and is associated with the prognosis of HCC patients. Further clinical data analysis shows that HNF4A-AS1 is an independent risk factor for the prognosis of HCC. *In vivo* and *in vitro* experiments show that HNF4A-AS1 suppresses the proliferation, metastasis, and stemness of HCC. Mechanistically, HNF4A-AS1 was found to inhibit the stability of AGR2 mRNA by promoting the proteasomal degradation of PCBP2, thereby participating in the regulation of the malignant phenotype of HCC. This study identified a potential target for the diagnosis and treatment of HCC.

## Materials and methods

### Human tissues and cell lines

The HCC tissues and adjacent non-cancerous tissues used in this study were obtained from the Hepatobiliary Centre of the First Affiliated Hospital of Nanjing Medical University. Prior to collection, written consent was obtained from all patients. The Ethics Committee at the First Affiliated Hospital of Nanjing Medical University granted approval for this study.

HCC cell lines, including Huh7, MHCC97H, HepG2, Hep3B, HCCLM3, SK-Hep1, and YY8103, the human embryonic kidney cell line 293T (HEK-293T), as well as the normal human liver cell line THLE-3, were obtained from the Shanghai Institute of Cell Biology, Chinese Academy of Sciences (Shanghai, China).

### Cell culture and transfection

We used Dulbecco's modified Eagle's medium (DMEM) (Gibco, CA, USA), supplemented with 10% fetal bovine serum (Gibco) and 50 U/ml penicillin-streptomycin (Invitrogen, CA, USA) for all cell cultures in a 37°C incubator with 5% CO2.

According to the provided protocol, plasmid and siRNA transfections were carried out using Lipofectamine 3000 (Invitrogen). Transfection efficiency was assessed through qRT-PCR and western blot analysis. For lentiviral transfections, interfering and plasmid sequences with high transfection efficiency were incorporated into lentivirus, and polybrene (Invitrogen) was utilized to enhance transfection efficiency. Puromycin (Invitrogen) was employed for selecting stable transfected cells. Lentivirus, siRNA, and plasmids were provided by GenePharma (Shanghai, China). The corresponding siRNA and shRNA sequences used in this experiment are listed in Supplementary Table 1.

### Quantitative real-time PCR (qRT‒PCR)

The total RNA extraction was performed using the RNA extraction kit (Invitrogen). The concentration of RNA was determined using a NanoDrop 2000 spectrophotometer (NanoDrop Technologies, MA, USA). The extracted RNA was then reverse transcribed into cDNA using the Prime Script RT kit (TaKaRa, Dalian, China). Quantitative real-time polymerase chain reaction (qRT-PCR) was performed using the SYBR Premix ExTaq II (TaKaRa) and the ABI 7900 PCR system (Applied Biosystems, CA, USA). The specific primer sequences used in the experiment can be found in Supplemental Table 2.

### Subcellular fractionation assays

The subcellular fractionation assay was conducted to evaluate nucleoplasmic separation using a Subcellular Fractionation Kit (Invitrogen) following the recommended protocols. In summary, the fractionation buffer was added to the cells and incubated on ice for 10 minutes. The mixture was subsequently centrifuged at 500 g and 4 °C for 5 minutes, resulting in the separation of the cytoplasm and nucleus. The nuclear fraction was then mixed with Cell Disruption Buffer and vortexed for 5 minutes. RNA extraction was carried out using an RNA extraction kit to isolate RNA from both the cytoplasmic and nuclear fractions.

### Western blot analysis

A solution of Radioimmunoprecipitation (RIPA) buffer containing 1 mM phenylmethylsulfonyl fluoride (PMSF) was prepared to extract total proteins. The proteins were then isolated using sodium dodecyl sulfate-polyacrylamide gel electrophoresis (SDS-PAGE) and transferred onto polyvinylidene fluoride (PVDF) membranes (Bio-Rad, CA, USA). The membranes were blocked with bovine serum albumin (BSA, 5%) for 2 hours. This was followed by overnight incubation with primary antibodies at 4 °C, and subsequent incubation with secondary antibodies at room temperature for 2 hours. Protein quantification was performed using hypersensitive ECL exposure solutions and Image Lab software (Bio-Rad, Hercules, CA, USA). The specific antibodies used in this experiment can be found in Supplemental Table 3.

### Agarose gel electrophoresis

In this study, DNA loading buffer was added to both the PCR amplification products and the T7 amplified RNA reverse transcription products. A 1X TBE solution (Beyotime) was used to prepare a 1% agarose solution. The amplified products were then subjected to electrophoresis in an agarose gel at a voltage of 100 V, and the position of the resulting bands was subsequently examined.

### Fluorescence *in situ* hybridization (FISH)

The design and synthesis of HNF4A-AS1, U6, and 14S FISH probes were completed by RiboBio (Guangzhou, China), and the FISH experiment was conducted using the Fluorescent Situ Hybridization Kit (RiboBio). A subset of cells was pretreated with 10 μg/ml of RNase A (Beyotime) for 2 hours at 37°C as a negative control. Then, the cells were blocked with prehybridization solution and incubated with 100 µl of hybridization solution containing the HNF4A-AS1, U6, and 14S probes overnight at 37°C. Images were acquired using a confocal laser microscope (Stellaris STED, Leica, Frankfurt, Germany).

### *In situ* hybridization (ISH) staining

The HCC tissues and adjacent non-cancerous tissues were obtained from the Hepatobiliary Centre of the First Affiliated Hospital of Nanjing Medical University. Human HCC tissue microarrays (TMAs) were created by Servicebio (Wuhan, China). After soaking the TMAs in xylene and anhydrous ethanol to remove wax, they are rehydrated in a gradient ethanol solution. Tissue is digested using pepsin, followed by dehydration in a gradient of ethanol. The design and synthesis of HNF4A-AS1 FISH probes were completed by RiboBio. The TMAs were blocked with prehybridization solution for 30 minutes and then incubated with 100 µl of hybridization solution containing the HNF4A-AS1 probes overnight at 37°C. The TMAs are then washed at 50 degrees Celsius with a gradient SSC solution, and the nuclei are stained with DAPI for 10 minutes.

### Cell counting kit-8 (CCK-8) assay

In this experiment, transfected cells were introduced into individual wells of a 96-well plate at a density of 1000 cells per well. Subsequently, the cells were incubated for 2 hours with 10 µl of CCK8 solution per well. The absorbance of each group was then measured at a wavelength of 450 nm over five consecutive days.

### Wound-healing assay

Transfected cells were cultured in a 6-well plate until they formed a monolayer covering the entire bottom of each well. To create a wound on the cell monolayer, a 200-µL pipette tip was used. The process of wound healing was observed and recorded at both 0 and 48 hours.

### Transwell assay

In the migration assay, a total of 2x10^4^ transfected cells were introduced into the upper chambers of Transwell plates, which contained 200 µl of serum-free medium. The lower chambers were filled with 400 µl of complete medium. Following a 48-hour incubation period, the cells were stained with crystal violet. The cells that remained in the upper chamber were discarded, and microscopic imaging was performed. In the invasion assay, the same procedures as the migration assay were followed, with the exception that 50 µl of Matrigel (BD Biosciences, NJ, USA) was added to the upper chamber.

### 5-Ethynyl-20-deoxyuridine (EdU) assay

The EdU assay was performed using an EdU Kit (Ribobio). Cells were seeded into a 24-well plate and incubated with an EdU solution for 2 hours. After fixation with 4% paraformaldehyde, cell permeabilization was carried out using Triton X-100. The Apollo solution was then used for cell staining. The nucleus was labeled with DAPI. Images were captured with a fluorescence microscope (DM4000B-1, Leica, Frankfurt, Germany).

### Sphere-forming assay

In this experiment, a 24-well suspension cell culture plate (Corning, NY, USA) was used to inoculate a total of 500 cells. These cells were then cultured for a period of 10 days in 2 mL of stem cell medium. The stem cell medium consisted of DMEM/F12 (Gibco) supplemented with 1X B27 (Sigma-Aldrich, MO, USA), 20 ng/mL EGF (PeproTech, NJ, USA), 20 ng/mL FGF (PeproTech), and 4 µg/mL heparin. Throughout the culture period, the number and size of the cell pellets were carefully observed and recorded.

### HCC organoid model

HCC tissue was obtained from the Hepatobiliary Centre of the First Affiliated Hospital of Nanjing Medical University, as mentioned previously. The specimens were fragmented into small pieces and then treated with collagenase D (Sigma‒Aldrich) for a duration of 2-4 hours at room temperature. The cells were then combined with BME (R&D Systems, MN, USA) on ice at a concentration of 10,000 cells in 40 µl of BME. Approximately 10 µl drops of BME were placed in each well of a 24-well suspension cell culture plate, followed by adding 400 µl of organoid medium to each well. The organoids were cultured in a 37°C incubator with 5% CO2, and the culture medium was replaced twice a week.

### Animal models

In this research, ethical approval for all animal experiments was obtained from the Institutional Animal Care and Use Committee (IACUC) at the First Affiliated Hospital of Nanjing Medical University. All procedures involving animals adhered to the operating guidelines set forth by the IACUC.

### Subcutaneous tumor model

BALB/c nude mice (4-week-old, male) were obtained from Vital River (Beijing, China) and were randomly assigned to four groups, each consisting of five mice. The transfected cells were suspended in PBS and then injected subcutaneously into the flanks of the mice. The size of the tumor was monitored every three days. After a four-week period, the mice were euthanized, and the volume and mass of the subcutaneous tumors were measured.

### Orthotopic xenograft model

The transfected cells were mixed with PBS at a concentration of 5×107/mL. The mice were disinfected and anesthetized. Subsequently, a small incision measuring 0.5 cm was made below the xiphoid process along the mid-abdominal line to expose the left lobe of the liver. A 20 µl cell suspension was injected beneath the capsule of the left liver. Cotton swabs were used to stop the bleeding for three minutes, and then the skin was stitched back together. After a four-week period, the intensity and distribution of fluorescence were observed, and the liver was analyzed.

### Lung metastasis model

The mice were grouped in the same manner as mentioned previously. The cells that were transfected were mixed with PBS (1×10^7^/mL) while on ice. Then, a suspension of 100 µL of cells was injected into the mice through the tail vein. After four weeks, the distribution and intensity of fluorescence were measured. The nude mice were then euthanized to quantify metastasis and perform immunohistochemistry.

### Primary hepatocellular carcinoma induction assay

Male C57 mice were divided into two groups, each consisting of 22 mice. On Day 14 after birth, the mice were administered a single dose of diethylnitrosamine (DEN, Sigma) at a concentration of 25 µg/g body weight via intraperitoneal injection. To upregulate the expression of the HNF4A-AS1 gene in the liver, an adeno-associated virus serotype 8 (AAV8) vector carrying the HNF4A-AS1 gene (HanBio, Shanghai, China) was injected into the mice's tail veins at a dosage of 4x10^10^ gene copies per mouse. The control group received an AAV8 vector carrying a control sequence. Two weeks after the DEN injection, the mice were given weekly intraperitoneal injections of CCL4 at a dosage of 0.5 µl/g of body weight for a total of 22 injections. After the final injection, ten mice from each group were sacrificed 48 hours later. The livers of the mice were collected, photographed, and then fixed for further analysis. The survival of the mice was also monitored, with a cut-off of 12 weeks, to determine their remaining survival time.

### Immunohistochemical (IHC) staining

Subcutaneous tumors, mouse liver, and mouse lung specimens were fixed in formaldehyde, embedded in paraffin, and subsequently sectioned. After dewaxing in xylene and absolute ethanol, the sections were rehydrated in a series of ethanol concentrations. Antigen retrieval was achieved by boiling the samples in a sodium citrate solution for 30 minutes. Blocking was performed using goat serum, and the primary antibody, appropriately diluted, was applied and allowed to incubate overnight at 4 degrees Celsius. Subsequently, the sections were washed with TBST, and the secondary antibody was added, then incubated at 37 degrees Celsius for 30 minutes. DAB chromogen (Beyotime) was used for staining, with hematoxylin employed for nuclear staining, followed by dehydration in a series of ethanol concentrations, clearing in xylene, and mounting of the slides.

### Pull-down assay

The full-length, antisense, and truncated forms of HNF4A-AS1 were provided by RiboBio. A pull-down assay was conducted using a pull-down kit (Thermo Scientific, USA). Magnetic beads were used to immobilize the RNAs, which were then mixed with RNA-binding buffer and incubated at room temperature for 30 minutes. The supernatant was removed, and the resulting precipitate was incubated with the cell lysate at 4°C for 1 hour. Protein extraction was performed using an elution buffer, and the bound proteins were detected using Western blot analysis.

### Silver staining assay and mass spectrometry analysis

Silver staining was performed using the Fast Silver Stain Kit (Beyotime) following established protocols. Mass spectrometry (MS) analysis was conducted by the Beijing Genomics Institute (GuangZhou, China).

### RNA immunoprecipitation (RIP)

An RNA immunoprecipitation kit (Geneseed, Guangzhou, China) was used to conduct RIP experiments following the provided protocols. In summary, the cell lysate was incubated overnight with magnetic beads and either PCBP2 antibody or anti-flag antibody. Subsequently, protease K was applied to the magnetic beads to eliminate the proteins. Finally, qRT-PCR was used to evaluate the isolated RNA.

### Coimmunoprecipitation (Co-IP) assay

In this experiment, cellular lysates, biotin-affinity agarose beads (Beyotime), and antibodies were subjected to overnight incubation at a temperature of 4°C. Following this, the agarose beads were washed five times with phosphate-buffered saline (PBS), and the protein was extracted using RIPA buffer.

### Bimolecular fluorescence complementation (BiFC) assay

The cDNA of PCBP2 and AIP4 was cloned into the BiFC vectors pBiFC-VN173 and pBiFC-VC155 (Addgene, MA, USA), respectively. The resulting recombinant plasmids were co-transfected into hepatocellular carcinoma (HCC) cells using Lipofectamine 3000. After 48 hours, the cells were fixed with 4% paraformaldehyde, stained with DAPI for 10 minutes, and imaged using a confocal laser microscope (Stellaris STED, Leica, Frankfurt, Germany).

### Ubiquitination detection assay

Transfected cells were treated with 20 μM MG132 (Beyotime) for 6 hours. After that, total protein was collected, and the lysates were pre-cleared with IgG and 30 μl of protein A + G Agarose (Beyotime) for 2 hours. The supernatant was harvested and immunoprecipitated using an anti-PCBP2 antibody. Western blotting was performed using an anti-Ubiquitin antibody to detect the ubiquitination of PCBP2.

For ubiquitination detection in HEK-293T cells, HA-tagged ubiquitin plasmids and Flag-tagged PCBP2 plasmids were co-transfected into HEK-293T cells using Lipofectamine 3000 (Invitrogen). The supernatant was immunoprecipitated using an anti-Flag antibody. An anti-HA antibody was used to detect the ubiquitination of PCBP2.

### Immunofluorescence assay

Cells were cultured on a confocal plate and then fixed with paraformaldehyde. Permeabilization was achieved using Triton, followed by blocking with goat serum. The cells were then incubated overnight with primary antibodies and subsequently with a fluorescence secondary antibody for 1 hour. Nuclei were labeled using DAPI (Beyotime). The resulting images were captured using a confocal microscope.

### RNA sequencing

TRIzol was used for the lysis of HCC tissues and transfected SK-Hep1 cells. RNA sequencing was conducted by the Beijing Genomics Institute (Guangzhou, China).

### Statistical analysis

The data obtained from the experiment conducted in this study were analyzed using SPSS software version 20.0 (IBM, SPSS, Chicago, IL, USA) and GraphPad Prism version 8.0 (GraphPad, San Diego, CA, USA). Statistical analysis was performed to determine the differences between two groups using the Student's t-test. For comparisons among more than two groups, one-way analysis of variance was employed. The clinical characteristics were assessed using the chi-square test, and correlation analysis was conducted using the Spearman correlation test. Survival curves were generated using the Kaplan-Meier method. Univariate and multivariate analyses were conducted using Cox proportional hazards models. A statistically significant difference was defined as a p-value of less than 0.05.

## Results

### The expression of HNF4A-AS1 is decreased in HCC and is associated with a favorable prognosis

**Figure 1:**
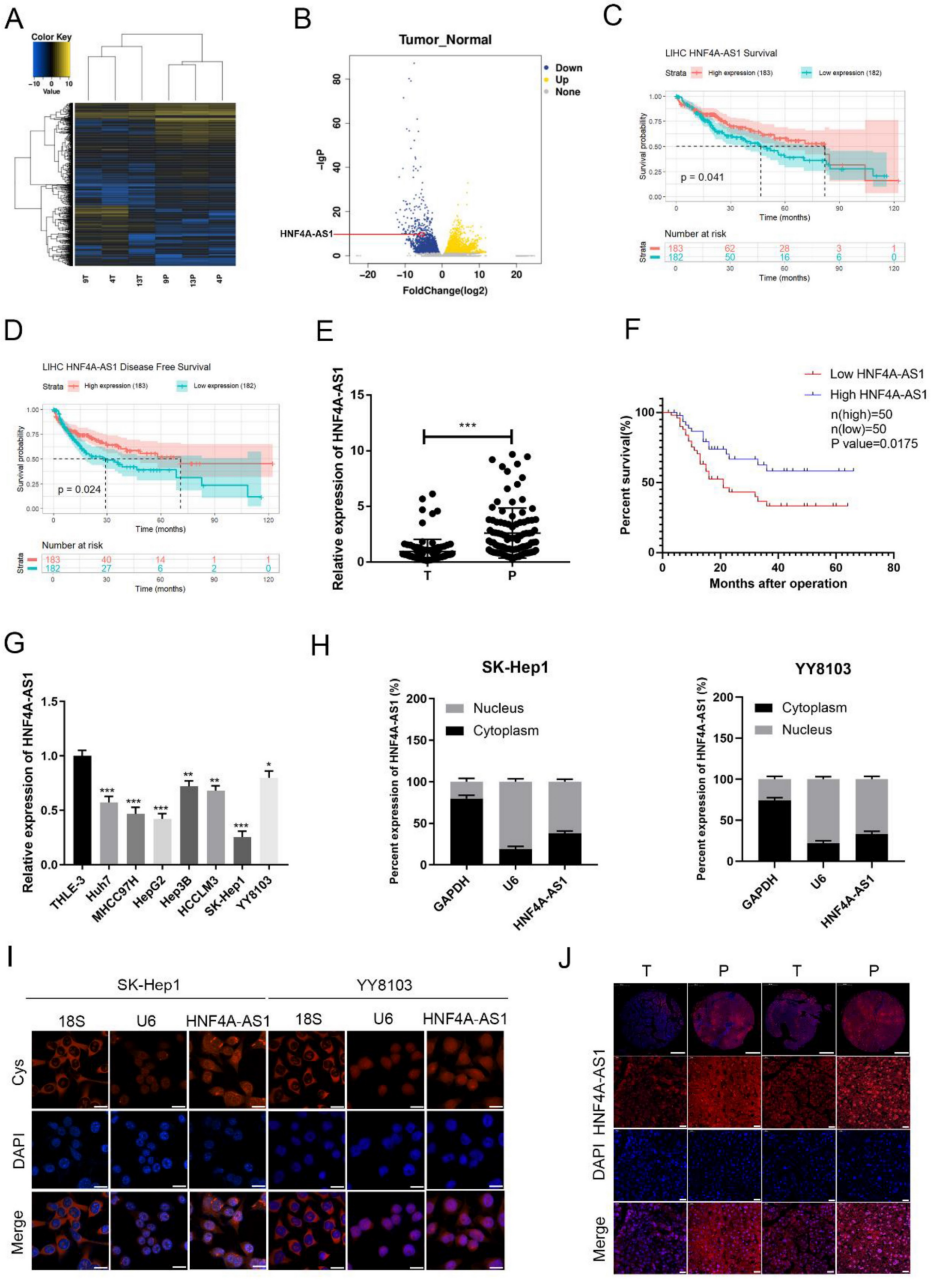
The expression of HNF4A-AS1 is decreased in HCC and is associated with a favorable prognosis. A. High-throughput sequencing heatmap of three pairs of HCC and adjacent carcinoma tissues (T: HCC tissue, P: para-carcinoma tissue). B. Volcano plot of differentially expressed lncRNAs between HCC and para-carcinoma tissues. C. OS curve of HCC patients with high or low HNF4A-AS1 expression (data from TCGA). D. Disease-free curve of HCC patients with high or low HNF4A-AS1 expression (data from TCGA). E. HNF4A-AS1 expression in 100 pairs of HCC tissues or para-carcinoma tissues was detected by qRT-PCR (T: HCC tissues, P: para-carcinoma tissues). F. Survival analysis of 100 HCC patients showed that the OS of patients with overexpressed HNF4A-AS1 was higher compared to the patients with low HNF4A-AS1 expression. G. HNF4A-AS1 expression in HCC cell lines and an immortalized liver cell line was detected by qRT-PCR. H. The relative expression level of HNF4A-AS1 in the nucleus/cytoplasm of SK-Hep1 and YY8103 cells was detected by a subcellular fractionation assay. I. FISH assay was used to measure the localization of HNF4A-AS1 in SK-Hep1 and YY8103 cells. Scale bar, 50 μm. J. ISH staining of HNF4A-AS1 in 2 pairs of HCC tissues cut from 43 pairs of HCC tissue chips. The original magnifications were 50× (scale bar, 200 μm) and 400× (scale bar, 50 μm). Bar graphs represent mean ± SEM (n=3, *P < 0.05, **P < 0.01, and ***P < 0.001).

To investigate lncRNAs associated with HCC, we conducted a high-throughput sequencing analysis to measure the expression levels of non-coding RNAs in both HCC and adjacent non-cancerous tissues (Figure 1A, B; Supplemental Table 4). Among the 263 differentially expressed lncRNAs (Log2FoldChange ≥ 5), a total of 139 were upregulated and 126 were downregulated in the HCC tissues compared to their paired normal tissues. According to sequencing analysis and the TCGA database survival analysis, 20 candidate lncRNAs were selected. The differential expression of these lncRNAs in 20 pairs of HCC tissues and adjacent non-cancerous tissues was assessed using qRT-PCR (Figure S1A). HNF4A-AS1 (NR_109949.1) was found to be significantly downregulated in tumor samples compared to adjacent non-cancerous tissues. HNF4A-AS1 is the antisense strand of the HNF4α gene, which has been shown to act as a tumor suppressor gene for HCC. Further analysis revealed that HNF4A-AS1 had minimal coding potential (Supplemental Table 5). Analysis of TCGA database shows that the expression of HNF4A-AS1 is positively correlated with the disease-free survival and overall survival of patients with HCC (Figure 1C, D). To evaluate the clinical significance of HNF4A-AS1 in HCC, we analyzed the expression level of HNF4A-AS1 in 100 HCC cases. The results showed that the expression of HNF4A-AS1 was decreased in HCC tissues (Figure 1E). Subsequently, we used the median value of the relative expression level as a cutoff to divide the patient cohort into high HNF4A-AS1 expression and low HNF4A-AS1 expression. Analysis of clinical data revealed that HNF4A-AS1 was associated with tumor size, TNM stage, and microvascular invasion (Table 1). Kaplan-Meier survival analysis demonstrated that patients with high levels of HNF4A-AS1 had higher overall survival rates in HCC (Figure 1F). Cox regression analysis confirmed that HNF4A-AS1 is an independent prognostic factor for HCC (Table 2). Additionally, the expression of HNF4A-AS1 was found to be decreased in HCC cell lines compared to normal liver cells (Figure 1G). We investigated the distribution of HNF4A-AS1 in SK-Hep1 and YY8103 cells using subcellular fractionation and FISH assays. The results showed that HNF4A-AS1 was distributed in both the cytoplasm and nucleus, and it was primarily localized in the nucleus of HCC cells (Figure 1H, I). We further used ISH staining to detect the expression and localization of HNF4A-AS1 in HCC tissue microarrays. The results showed that HNF4A-AS1 is downregulated in HCC and primarily localized in the cell nucleus (Figure 1J, Figure S1B).

### HNF4A-AS1 inhibits the metastasis and proliferation of HCC cells

**Figure 2:**
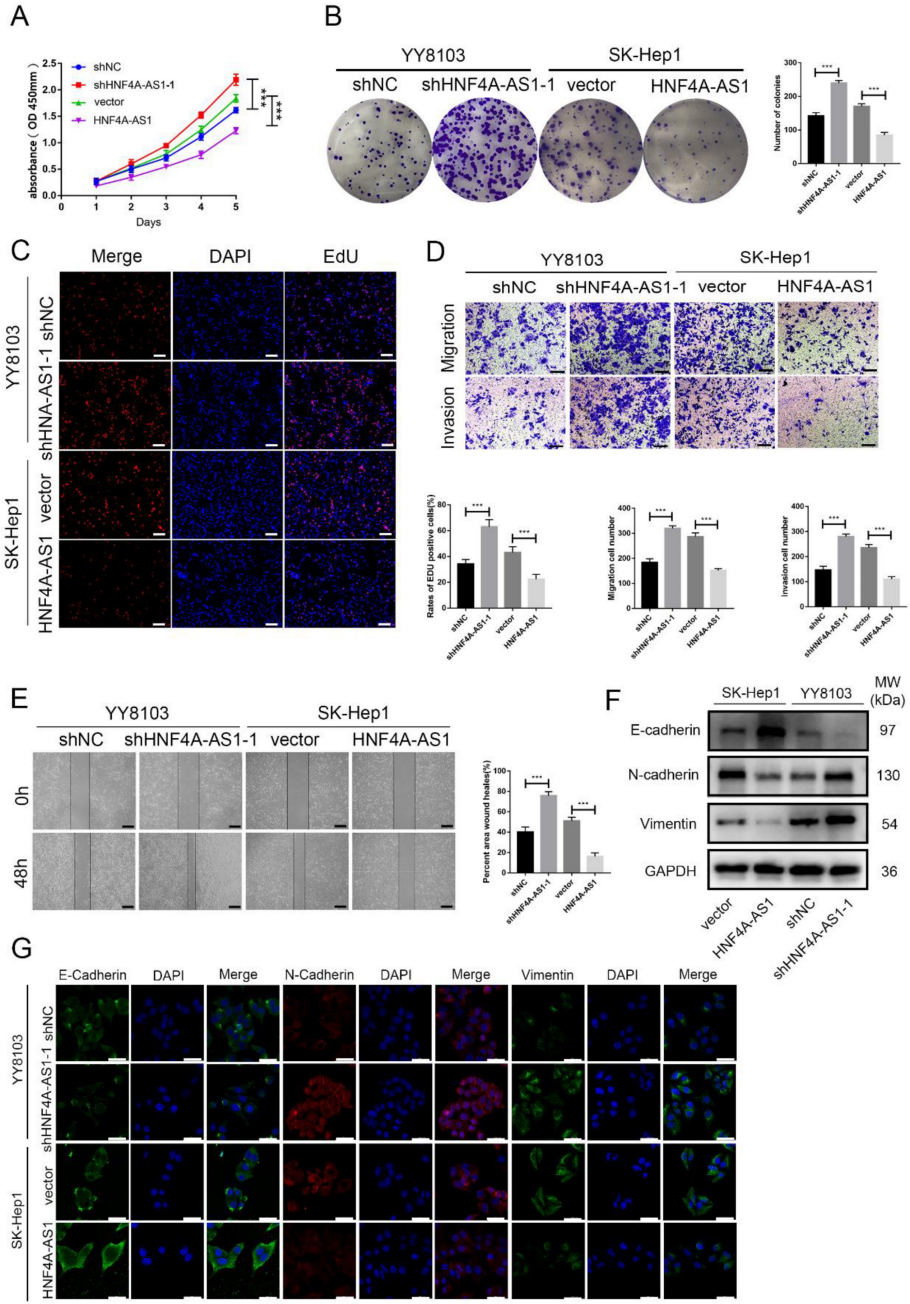
HNF4A-AS1 suppresses proliferation and metastasis of HCC cells. A. The effect of HNF4A-AS1 overexpression and knockdown on the proliferation of HCC cells was assessed using a CCK8 assay. B-C. A colony formation assay (B) and EdU assay (C) presented that HNF4A-AS1 knockdown promoted the proliferation of YY8103 cells, and that overexpression of HNF4A-AS1 suppressed SK-Hep1 cell proliferation. Scale bar, 50 μm. D. Transwell assays showed that knockdown of HNF4A-AS1 promoted cell invasion and migration in YY8103 cells, whereas HNF4A-AS1 overexpression suppressed the migration and invasion capacities of SK-Hep1 cells. Scale bar, 200 μm. E. A wound-healing assay was used to determine the role of knockdown and overexpression of HNF4A-AS1 in the motility of HCC cells. Scale bar, 500 μm. F-G. EMT-associated proteins were measured by western blot assay (F) and immunofluorescence (G) in HCC cells. Scale bar, 50 μm. Bar graphs represent mean ± SEM (n=3, ***P < 0.001).

Based on the varying levels of HNF4A-AS1 in different HCC cell lines, we used SK-Hep1 cells with low HNF4A-AS1 levels to overexpress HNF4A-AS1 through lentiviral infection. Conversely, we utilized YY8103 cells with high expression of HNF4A-AS1 to knock down HNF4A-AS1 using a lentivirus carrying shRNA. The effectiveness of HNF4A-AS1 knockdown and overexpression was confirmed through qRT-PCR (Figure S2A). Our results from EdU, colony formation, and CCK-8 assays demonstrated that knocking down HNF4A-AS1 promoted YY8103 cell proliferation, while overexpressing HNF4A-AS1 suppressed SK-Hep1 cell proliferation (Figure 2A-C, Figure S2B-D). Wound-healing and transwell assays revealed that knocking down HNF4A-AS1 increased the metastasis of YY8103 cells, while overexpressing HNF4A-AS1 reduced the metastasis of SK-Hep1 cells (Figure 2D, E, Figure S2E-F). Studies have shown that the migration and invasion abilities of tumor cells are closely related to the process of epithelial-mesenchymal transition (EMT) process^19^. We examined the proteins associated with EMT through Western blot analysis and immunofluorescence assays. Overexpressing HNF4A-AS1 was found to decrease the expression of N-cadherin and vimentin, while increasing the expression of E-cadherin. These effects were reversed upon knockdown of HNF4A-AS1 (Figure 2F, G). Overall, our findings indicate that HNF4A-AS1 inhibits cell proliferation, invasion, migration, and suppresses the EMT process in HCC cells.

### HNF4A-AS1 suppresses the stemness of HCC cells

**Figure 3:**
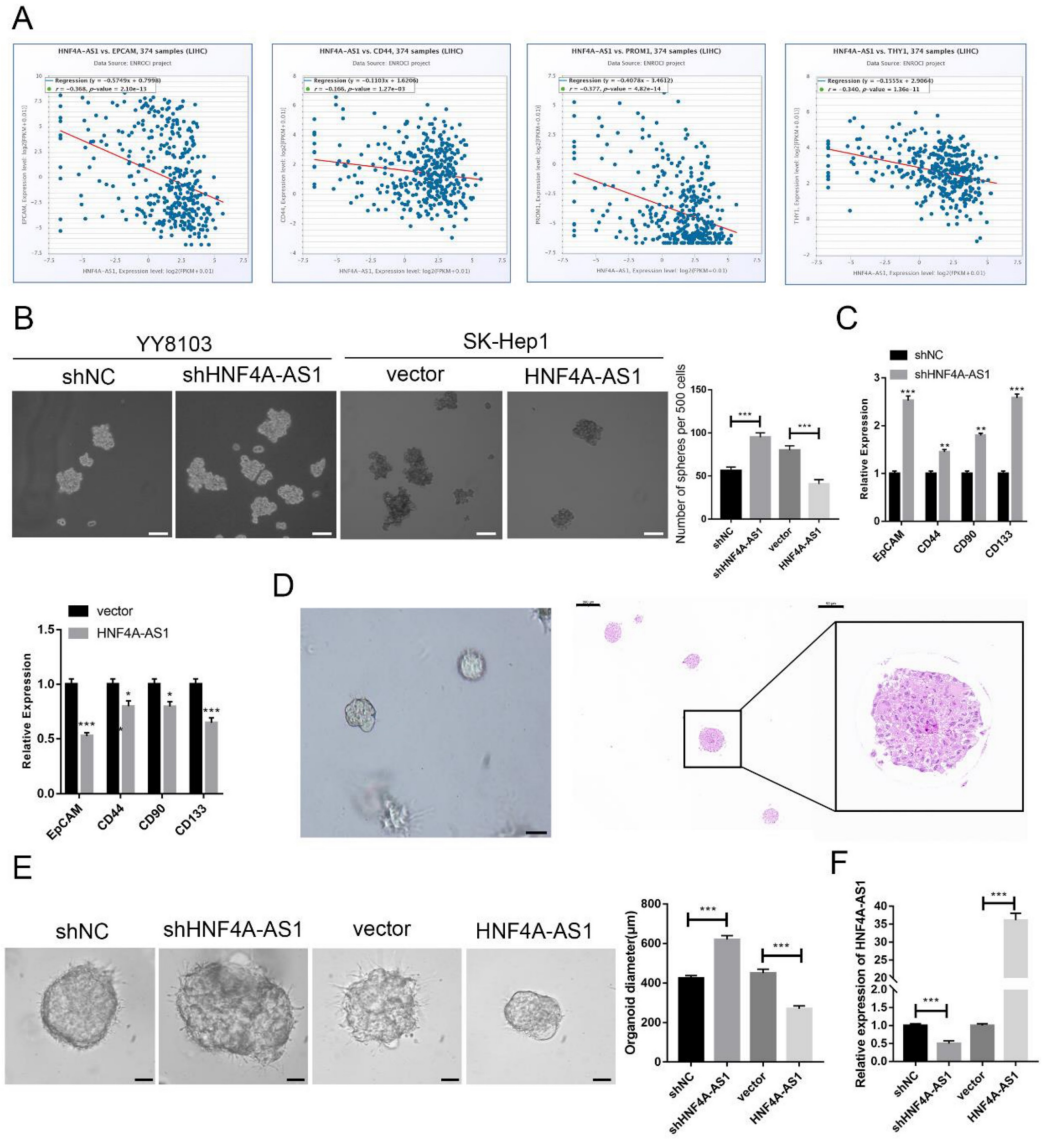
HNF4A-AS1 suppresses the stemness of HCC cells. A. HNF4A-AS1 was negatively associated with EPGAM, CD44, PROM1 (CD90), and THY1 (CD133) (data from starBase). B. Sphere-forming assays showed that spheroid formation was enhanced in HNF4A-AS1-deficient YY8103 cells but attenuated in HNF4A-AS1-overexpressing SK-Hep1 cells. Scale bar, 100 μm. C. The expression levels of CSC-related biomarkers (EpCAM, CD133, CD44, and CD90) in spheroids were detected by qRT-PCR. D. Photographs (left) and HE staining (right) of HCC organoids. Scale bar, 200 μm (left), 200 μm (middle), 50 μm (right). E. HNF4A-AS1 expression in organoids was altered by a lentivirus carrying shRNA or overexpression plasmid, and organoid diameter was measured after 7 days of culture. Scale bar, 50 μm. F. The knockdown and overexpression efficiencies of HNF4A-AS1 in organoids were measured by qRT-PCR. Bar graphs represent mean ± SEM (n=3, *P < 0.05, **P < 0.01 and ***P < 0.001).

HCC is heterogeneous and contains cells with stem-like properties. Cancer stem cells (CSCs) are believed to play a role in the initiation and progression of HCC^20^-^22^. The analysis of the starBase database revealed a negative correlation between the expression of HNF4A-AS1 and CSC-related biomarkers (EPCAM, CD44, PROM1, and THY1) in HCC samples (Figure 3A). This suggests that HNF4A-AS1 may inhibit the stemness of HCC. The enrichment of CSCs from HCC cell lines through sphere-forming assays indicated that HNF4A-AS1-deficient YY8103 cells exhibited enhanced spheroid formation, while SK-Hep1 cells overexpressing HNF4A-AS1 showed attenuated spheroid formation (Figure. 3B, Figure S2G). qRT-PCR analysis of CSC-related biomarkers (EpCAM, CD133, CD44, and CD90) demonstrated that silencing HNF4A-AS1 resulted in an upregulation of these biomarkers. Conversely, the overexpression of HNF4A-AS1 led to a downregulation of their expression (Figure 3C, Figure S2H). Additionally, three-dimensional organoids derived from patients with HCC were used to simulate the clinical environment (Figure 3D). Lentiviruses carrying shRNA or overexpression plasmids were utilized to modify the expression of HNF4A-AS1 in the organoids, and the transfection efficiency was confirmed using qRT-PCR (Figure 3F). Knockdown of HNF4A-AS1 resulted in larger tumor organoids, while overexpression of HNF4A-AS1 led to smaller spheroid structures (Figure 3E). These findings collectively indicate that HNF4A-AS1 suppresses the stemness of HCC.

### HNF4A-AS1 inhibits tumor growth and metastasis *in vivo*

**Figure 4:**
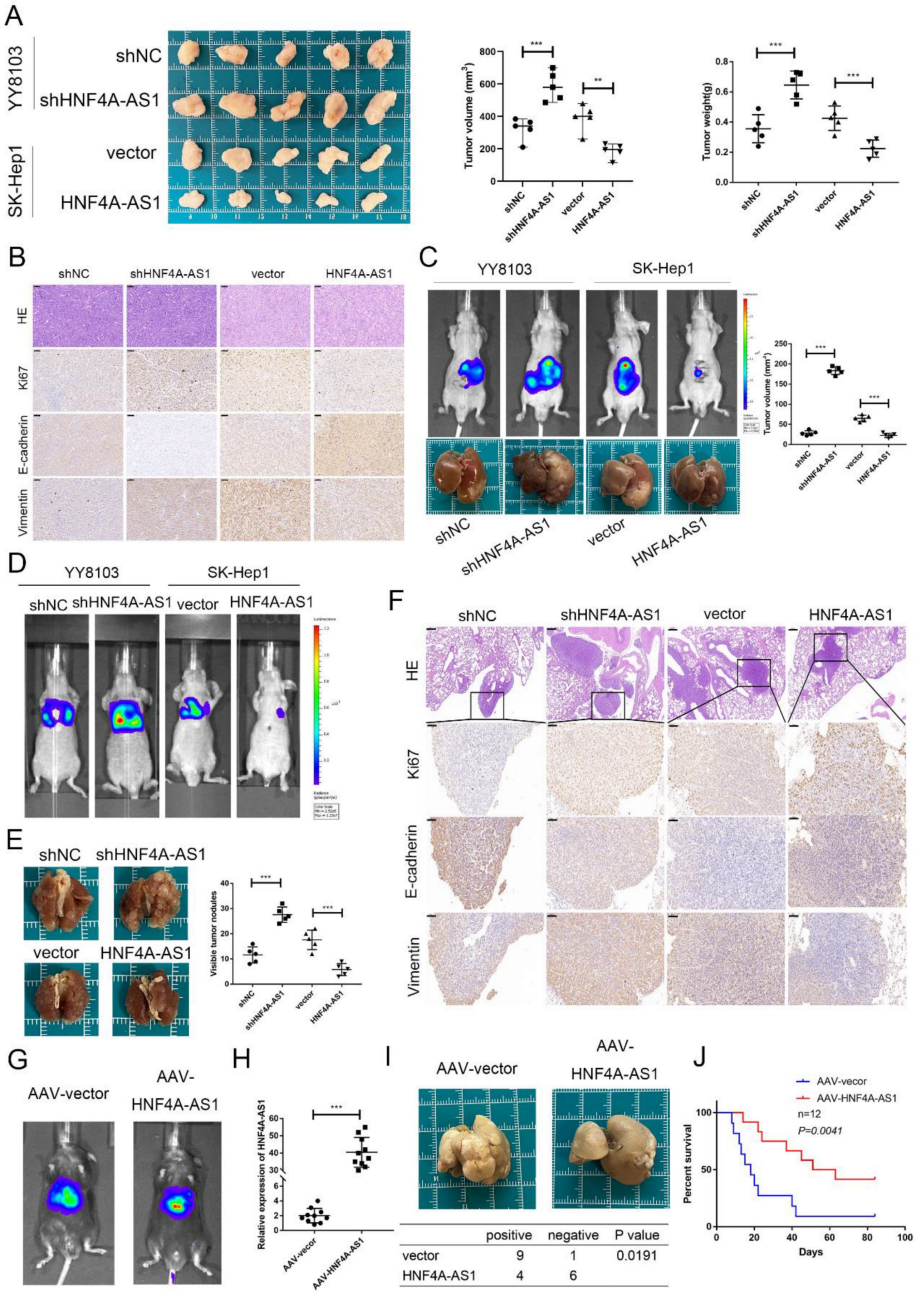
HNF4A-AS1 inhibits tumor growth and metastasis *in vivo*. A. A subcutaneous tumor model was utilized to detect the proliferation of HNF4A-AS1-deficient YY8103 cells and HNF4A-AS1-overexpressing SK-Hep1 cells *in vivo*. Photograph of subcutaneous tumors (left), growth curve of subcutaneous tumors (middle), and weight of subcutaneous tumors (right). B. Immunohistochemistry of HE, Ki67, EpCAM, and CD133 in subcutaneous tumors. Scale bar, 50 μm. C. An orthotopic xenograft model showed that HNF4A-AS1 inhibited HCC cell proliferation *in vivo*. Lentivirus carrying luciferase tag and *in vivo* image of nude mice (above), representative images of livers (below), and transplanted tumor volume (left). D. *In vivo* imaging of a lung metastasis model in nude mice. E. The representative images of lungs. F. Immunohistochemistry of HE (Scale bar, 200 μm) and EMT-related proteins in metastases (Scale bar, 50 μm). G. AAV-8 carrying the HNF4A-AS1 overexpression plasmid and luciferase tag was administered to mice via tail vein injection. *In vivo* image of mice. H. The efficiency of transfection with AAV-8 was assessed by qRT-PCR. I. Representative images of livers of DEN-induced spontaneous HCC model (above) and HCC induction statistics (below). J. Survival curves of HNF4A-AS1 overexpression and control mice. Bar graphs represent mean ± SEM (n=3, **P < 0.01 and ***P < 0.001).

In order to assess the role of HNF4A-AS1 in HCC, a series of *in vivo* models were established. The findings from the subcutaneous tumor model demonstrated that the volume and weight of YY8103 tumors increased when HNF4A-AS1 was knocked down, whereas the volume and weight of SK-Hep1 tumors decreased when HNF4A-AS1 was overexpressed (Figure 4A). Immunohistochemical staining of Ki67, vimentin, E-cadherin, EpCAM, and CD133 (THY1) in the xenograft tumors indicated that HNF4A-AS1 suppressed cell proliferation, stemness, and the EMT pathway *in vivo* (Figure 4B, Figure S2I). The functional role of HNF4A-AS1 was further elucidated *in vivo* by creating an orthotopic xenograft model. Tumor growth was significantly inhibited in the group with overexpressed HNF4A-AS1, while the opposite effect was observed in the group with HNF4A-AS1 knockdown (Figure 4C).

The metastasis of HCC cells was evaluated using a lung metastasis model. Histological examination and bioluminescence imaging of the lung metastasis model revealed that HNF4A-AS1 knockdown promoted lung metastasis, whereas overexpression of HNF4A-AS1 suppressed lung metastasis (Figure 4D, E). Immunohistochemical staining of markers such as Ki67, E-cadherin, and vimentin in the lung metastases further confirmed the inhibitory effect of HNF4A-AS1 on the EMT signaling pathway *in vivo* (Figure 4F).

We constructed DEN- and CCL4-induced spontaneous hepatocellular carcinoma models to investigate the role of HNF4A-AS1 in the initiation and progression of HCC, as well as its potential therapeutic value for HCC. To establish the models, mice were intraperitoneally injected with DEN at 3 weeks of age. Subsequently, an adeno-associated virus serotype 8 (AAV-8) carrying the HNF4A-AS1 overexpression plasmid was injected into the tail vein to specifically target hepatic HNF4A-AS1. The transfection efficiency was assessed using *in vivo* imaging and qRT-PCR (Figure 4G, H). Four weeks after DEN injection, CCL4 was intraperitoneally administered once a week. Our results showed that the incidence of tumors was significantly reduced in the group overexpressing HNF4A-AS1 compared to the control group (Figure 4I). Furthermore, survival analysis revealed a prolonged overall survival in the group with HNF4A-AS1 overexpression compared to the control group (Figure 4J). These findings suggest that HNF4A-AS1 inhibits the occurrence and progression of HCC, indicating its potential as a therapeutic target for HCC treatment.

### HNF4A-AS1 physically interacts with PCBP2 in HCC cells

**Figure 5:**
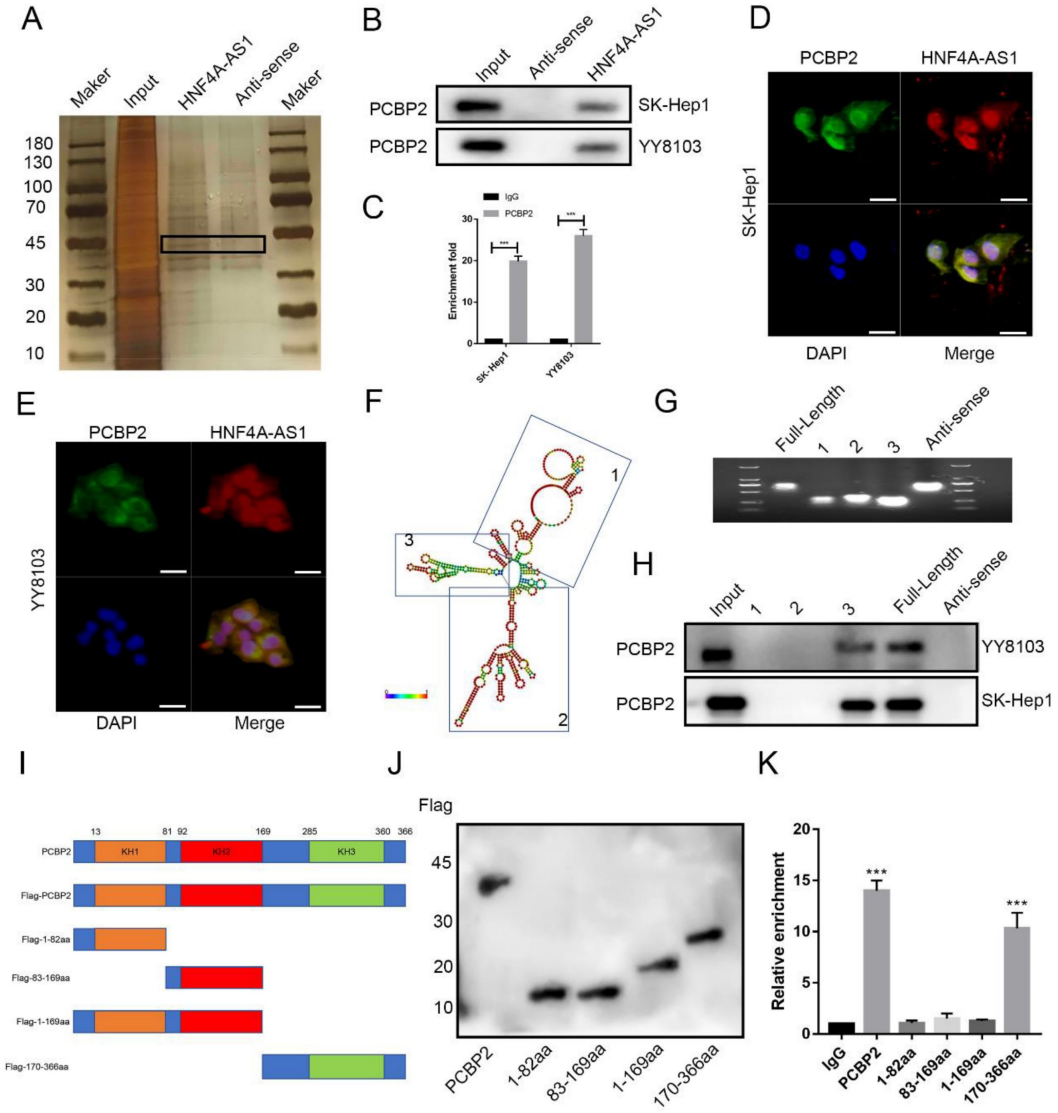
HNF4A-AS1 physically interacts with PCBP2 in HCC cells. A. Silver staining was performed with the sense and antisense HNF4A-AS1 pull-down proteins. The specific bands were marked with arrows. B. Western blot was carried out to determine PCBP2 in the sense and antisense HNF4A-AS1 pull-down proteins. C. RIP assay showed that HNF4A-AS1 was enriched by an anti-PCBP2 antibody. D-E. Immunofluorescence showed that PCBP2 colocalized with HNF4A-AS1 in SK-Hep1 (D) and YY8103 (E) cells. Scale bar, 50 μm. F. Secondary stem-loop structure of HNF4A-AS1 and truncated fragments. G. Agarose gel electrophoresis analysis of full-length HNF4A-AS1, antisense HNF4A-AS1, and truncated fragments of HNF4A-AS1. H. Western blot was conducted to detect PCBP2 in the pull-down proteins of full-length HNF4A-AS1, antisense HNF4A-AS1, and truncated fragments of HNF4A-AS1. I. PCBP2 was truncated (1-82 aa, 83-169 aa, 1-169 aa, and 170-366 aa) according to its protein domains. J. Truncated constructs were tagged with Flag, and western blot was performed to determine transfection efficiency. K. The enrichment of HNF4A-AS1 in cells transfected with full-length and truncated Flag-tagged constructs was detected via RIP assays. Bar graphs represent mean ± SEM (n=3, and ***P < 0.001).

The regulation of tumor progression through the binding of RNA-binding proteins (RBPs) is a significant mechanism of lncRNAs. In order to investigate the mechanism of HNF4A-AS1 in HCC, a pulldown assay was conducted to identify the binding proteins of HNF4A-AS1. The full-length sequence of HNF4A-AS1 was obtained through T7 amplification *in vitro*, with the antisense chain serving as a control. Silver staining analysis revealed specific bands in the sense group, indicating the presence of potential binding proteins (Figure 5A). Mass spectrometry analysis was then performed to identify the binding proteins of HNF4A-AS1. The antisense pull-down products were excluded to obtain the sense-specific binding proteins (Figure S3A), and GO enrichment analysis was performed for the sense-specific binding proteins (Figure S3B). The top 20 proteins were selected for further analysis (Supplemental Table 6). The results of the silver staining assays showed that a specific band was observed at 45 kDa, and PCBP2 was the only one among the 20 candidate binding proteins that had the required molecular weight (Figure S3C). PCBP2, an RNA-binding protein, plays a crucial role in regulating cancer-associated biological processes. Data from starBase showed that PCBP2 was overexpressed in HCC (Figure S4A). Kaplan-Meier survival analysis demonstrated that patients with HCC who had low levels of PCBP2 exhibited increased disease-free survival and overall survival compared to those with high levels of PCBP2 (Figure S4B, C). Based on these findings, it was hypothesized that PCBP2 is one of the RBPs that specifically interacts with HNF4A-AS1 in HCC. Immunoblotting of the proteins pulled down by HNF4A-AS1 and its antisense chain confirmed that PCBP2 was a direct binding partner of HNF4A-AS1 (Figure 5B). Consistently, RNA immunoprecipitation assays demonstrated that HNF4A-AS1 was significantly enriched by the anti-PCBP2 antibody (Figure 5C). Additionally, immunofluorescence results showed that HNF4A-AS1 colocalized with PCBP2 in HCC cells (Figure 5D, E).

To determine the binding site of HNF4A-AS1 and PCBP2, we constructed various deletion mutants of HNF4A-AS1 based on its secondary stem loop structure (Figure 5F) and the predicted binding site of PCBP2 on HNF4A-AS1 (Figure S3D). The deletion mutant sequences (Figure S3E) were verified by agarose gel electrophoresis (Figure 5G). Based on the RNA pull-down results, it was found that fragment 3 (491-672 nt) of HNF4A-AS1 mediated its interaction with PCBP2 (Figure 5H). PCBP2 contains three KH domains (KH1-3) that recognize and bind RNA. Therefore, full-length and truncated versions of PCBP2 were constructed to identify the binding domain of PCBP2 to HNF4A-AS1 (Figure 5I, J). RNA immunoprecipitation assays revealed that the KH3 domain (285-360 aa) of PCBP2 contributed to its interaction with HNF4A-AS1 (Figure 5K).

### HNF4A-AS1 mediates the proteasomal degradation of PCBP2

**Figure 6:**
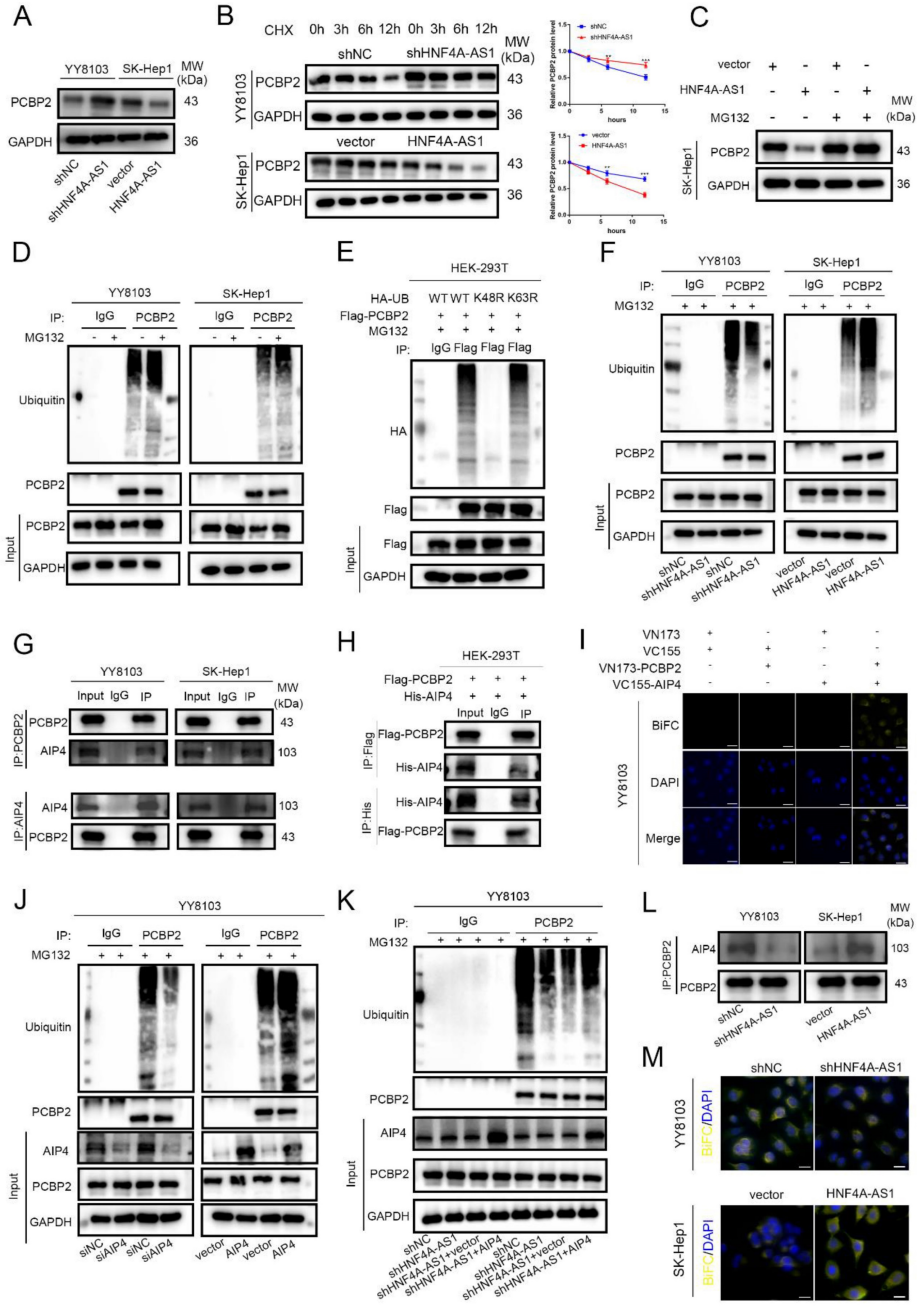
HNF4A-AS1 mediates the protease degradation of PCBP2. A. Western blot results exhibited that knockdown of HNF4A-AS1 promoted PCBP2 protein levels, while overexpression of HNF4A-AS1 suppressed the level of PCBP2. B. HNF4A-AS1-deficient YY8103 cells and HNF4A-AS1-overexpressing SK-Hep1 cells were treated with CHX (50 μg/mL) for the indicated times, and PCBP2 and GAPDH were measured by Western blot assay. C. Western blot analysis demonstrated that the decrease in PCBP2 protein due to HNF4A-AS1 overexpression was reversed by MG132 (20 μM, 12h). D. Western blot assay showed that PCBP2 ubiquitination was increased in the MG132 treatment group. E. Ubiquitination detection in HEK-293T cells co-transfected with HA-tagged ubiquitin plasmids and Flag-tagged PCBP2 plasmids revealed that the ubiquitination level in the K48R group was significantly lower than that in the WT group. F. The ubiquitination of PCBP2 was reduced in HNF4A-AS1-deficient YY8103 cells and increased in HNF4A-AS1-overexpressing SK-Hep1 cells. G. CoIP assay indicated that PCBP2 was enriched by an anti-AIP4 antibody and that AIP4 was enriched by an anti-PCBP2 antibody. H. Co-immunoprecipitation assay in HEK-293T cells co-transfected with Flag-tagged PCBP2 and His-tagged AIP4 plasmids showed that PCBP2 can directly interact with AIP4. I. The BiFC assay revealed that AIP4 and PCBP2 were bound in the YY8103 cells. Scale bar, 50 μm. J. Western blot analysis demonstrated that AIP4 promoted PCBP2 ubiquitination. K. AIP4 overexpression counteracted the suppressive effect of HNF4A-AS1 knockdown on PCBP2 ubiquitination. L-M. IP assay and BiFC assay showed that HNF4A-AS1 facilitated PCBP2 recruitment of AIP4. Scale bar, 50 μm. Bar graphs represent mean ± SEM (n=3, **P < 0.01 and ***P < 0.001).

The regulatory effect of PCBP2 on HNF4A-AS1 was investigated in this study. PCBP2 was knocked down using siRNA and overexpressed using an overexpression plasmid. The transfection efficiency was assessed by Western blot, and siPCBP2-1 was found to have the highest knockdown efficiency (Figure S5A). Therefore, it was used in subsequent experiments. qRT-PCR analysis showed no significant change in the abundance of HNF4A-AS1 with either PCBP2 knockdown or overexpression (Figure S5B, C). However, western blot analysis revealed that the expression of the PCBP2 protein was up-regulated in HNF4A-AS1-knockdown cells and decreased in HNF4A-AS1-overexpressing cells (Figure 6A). qRT-PCR analysis showed that the level of PCBP2 mRNA was not significantly affected by HNF4A-AS1 (Figure S5D). A protein stability assay using cycloheximide (CHX) demonstrated that PCBP2 was more stable when HNF4A-AS1 was knocked down and more easily degraded after HNF4A-AS1 overexpression (Figure 6B). This indicates that HNF4A-AS1 inhibits the stability of PCBP2. Western blot analysis showed that the downregulation of PCBP2 resulting from HNF4A-AS1 overexpression was ameliorated by a proteasomal inhibitor (MG132) (Figure 6C). Additionally, PCBP2 ubiquitination was upregulated after MG132 treatment (Figure 6D), indicating that HNF4A-AS1 may promote the proteasomal degradation of PCBP2 by regulating its ubiquitination.

In order to investigate the specific ubiquitin chains involved in the ubiquitination of PCBP2, we separately constructed wild-type, K48 mutant, and K63 mutant ubiquitin plasmids (WT, K48R, K63R). The HA-tagged ubiquitin plasmids were co-transfected with Flag-tagged PCBP2 plasmids into HEK-293T cells. The results of ubiquitination detection showed that the ubiquitination level in the K48R group was significantly lower than that in the WT group, while there was no significant difference between the K63R group and the WT group (Figure 6E). This indicates that the ubiquitination type of PCBP2 is K48-linked ubiquitination. The ubiquitination of PCBP2 was enhanced in cells overexpressing HNF4A-AS1 but decreased in HNF4A-AS1-knockdown cells (Figure 6F). This suggests that HNF4A-AS1 promotes the degradation of PCBP2 by facilitating its ubiquitination.

We further investigated the mechanism by which HNF4A-AS1 regulates the ubiquitination of PCBP2. AIP4 (ITCH) was identified as one of the proteins through RNA pull-down assays combined with mass spectrometry (Supplemental Table 6). Previous studies have demonstrated that PCBP2 can bind to the HECT domain-containing E3 ligase, AIP4^23^. Pull-down assays confirmed that AIP4 is a direct binding partner of HNF4A-AS1 (Figure S6A). Co-immunoprecipitation assay results indicated that PCBP2 directly interacts with AIP4 in HCC and HEK-293T cells (Figure 6G-H). Furthermore, the BiFC experimental results showed that only HCC cells co-transfected with VN-173-PCBP2 and VC-155-AIP4 exhibited fluorescence, indicating the binding of PCBP2 and AIP4 in HCC cells (Figure 6I, Figure S6B). Knockdown and overexpression of AIP4 were performed to investigate its modulatory role in the ubiquitination of PCBP2 in HCC. The transfection efficiency was assessed by Western blot, and siAIP4-1 was used in subsequent experiments (Figure S6C).

Western blot analysis showed that AIP4 promotes the ubiquitination of PCBP2 in HCC (Figure 6H, Figure S6D), indicating that AIP4 acts as the E3 ligase for PCBP2 in HCC. Knockdown of AIP4 rescued the promoting role of overexpressed HNF4A-AS1 in PCBP2 ubiquitination, and overexpression of AIP4 diminished the inhibitory effect of HNF4A-AS1 knockdown on PCBP2 ubiquitination (Figure 6I, Figure S6E). Immunoprecipitation results demonstrated that the upregulation of HNF4A-AS1 contributed to the recruitment of PCBP2 to AIP4. Conversely, the knockdown of HNF4A-AS1 inhibited the binding of PCBP2 to AIP4 (Figure 6K). Meanwhile, BiFC experiments demonstrated that reducing HNF4A-AS1 expression inhibited the interaction between PCBP2 and AIP4, whereas upregulation of HNF4A-AS1 enhanced the interaction between PCBP2 and AIP4 (Figure 6L). These findings indicate that HNF4A-AS1 enhances the binding of PCBP2 to AIP4, thereby facilitating the ubiquitination and subsequent degradation of PCBP2.

### HNF4A-AS1 suppresses the mRNA stability of ARG2 by downregulating PCBP2

**Figure 7:**
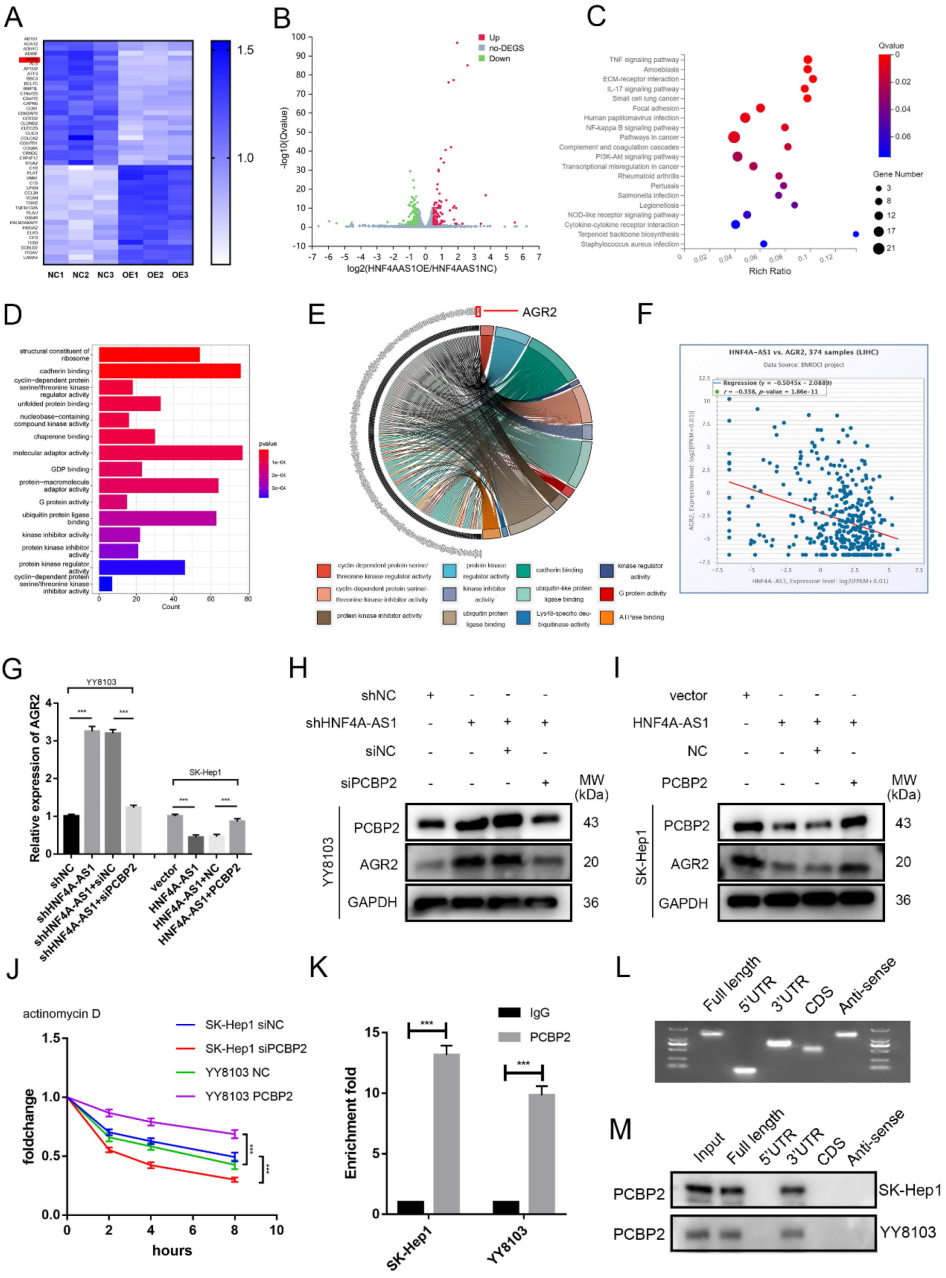
HNF4A-AS1 suppresses the mRNA stability of ARG2 through the downregulation of PCBP2. A-B. The RNA-seq heatmap (A) and volcano map (B) of three pairs of HNF4A-AS1-overexpressing SK-Hep1 cells and negative control cells (NC: negative control, OE: HNF4A-AS1-overexpressing). C. KEGG pathway analysis of the RNA-seq results. D. GO enrichment analysis of the RNA-seq results. E. Chord plot for GO enrichment analysis of the RNA-seq results. F. HNF4A-AS1 and AGR2 mRNA were negatively correlated in HCC tissues (data from starBase). G. qRT-PCR presented that the overexpression of HNF4A-AS1 inhibited the mRNA expression of AGR2, while the overexpression of PCBP2 counteracted the inhibitory effect of HNF4A-AS1 on AGR2, and vice versa. H-I. Western blot analysis demonstrated that knockdown of PCBP2 diminished the promotive effect of HNF4A-AS1 knockdown on AGR2 (H), and that PCBP2 overexpression restored the inhibiting impact of HNF4A-AS1 on AGR2 (I). J. PCBP2-deficient SK-Hep1 cells and PCBP2-overexpressing YY8103 cells were treated with actinomycin D for the indicated times, and AGR2 mRNA was detected by qRT-PCR. K. RIP assay showed that AGR2 mRNA was enriched by PCBP2. L. Full-length, antisense, 3'UTR, 5'UTR, and CDS fragments of AGR2 mRNA were transcribed and biotinylated *in vitro*, and agarose gel electrophoresis was performed to detect these fragments. M. An RNA pull-down assay was employed to identify the binding fragments of AGR2 mRNA with PCBP2. Bar graphs represent mean ± SEM (n=3, and ***P < 0.001).

In order to gain a better understanding of the mechanisms by which HNF4A-AS1 influences the progression of HCC cells, the researchers conducted RNA sequencing to investigate the transcriptional profiles of SK-Hep1 cells that were overexpressing HNF4A-AS1 (Figure 7A, B, Supplemental Table 7). The results of the KEGG pathway analysis revealed that the overexpression of HNF4A-AS1 negatively regulates NFκB, PI3K-AKT, and pathways in cancers (Figure 7C, Figure S7A). Additionally, the GO enrichment analysis indicated that HNF4A-AS1 plays a role in regulating cadherin, cyclin-dependent proteins, and ubiquitin protein ligase (Figure 7D). To further investigate the downstream targets of HNF4A-AS1, we performed a gene distribution analysis using GO enrichment analysis of the RNA-seq results. The chord plot revealed that AGR2 is implicated in the majority of pathways related to tumors (Figure 7E). Additionally, AGR2 emerged as one of the top-scoring genes in the RNA-seq results. Analysis of the starBase database showed a negative correlation between HNF4A-AS1 and AGR2 (Figure 7F). To investigate the regulatory role of AGR2 in HCC, we utilized siRNA and plasmid to knock down or overexpress AGR2 expression in HCC cells. The transfection efficiency of AGR2 was assessed using western blot analysis. siAGR2-2 was found to have the highest knockdown efficiency. Therefore, it was embedded into the lentivirus and used in subsequent experiments (Figure S7B). *In vitro* functional studies demonstrated that AGR2 promotes the proliferation, invasion, and stemness of HCC cells (Figure S7C-F).

Next, we investigated whether HNF4A-AS1 regulates AGR2 through PCBP2. Western blot and qRT-PCR results indicated that the overexpression of HNF4A-AS1 suppressed the expression of AGR2 mRNA and protein. Subsequent experiments demonstrated that the overexpression of PCBP2 restored the inhibitory effect of HNF4A-AS1 on AGR2, and vice versa (Figure 7G, H, I). Previous studies have demonstrated that PCBP2 binds to the 3' UTR of mRNA and maintains mRNA stability^24^. The mRNA stability assay using actinomycin D revealed that the knockdown of PCBP2 decreased the stability of AGR2 mRNA, while the overexpression of PCBP2 had the opposite effect (Figure 7J). Additionally, RIP assays demonstrated that AGR2 mRNA was significantly enriched with the anti-PCBP2 antibody (Figure 7K). The full-length, 3'UTR, 5'UTR, and CDS sequences of AGR2 mRNA were obtained from the UCSC database (Figure S8A). These fragments were then constructed *in vitro* using the T7 amplification assay. The amplified sequences were analyzed using agarose gel electrophoresis (Figure 7L). The results of the RNA pull-down experiment indicate that the full-length AGR2 mRNA and the 3' UTR fragment are involved in the interaction between AGR2 mRNA and PCBP2 (Figure 7M). Taken together, these findings suggest that PCBP2 maintains the stability of AGR2 mRNA by binding to the AGR2 mRNA 3' UTR, and HNF4A-AS1 reduces AGR2 levels by promoting the degradation of PCBP2.

### HNF4A-AS1 inhibits the progression of HCC through the PCBP2/AGR2 axis

**Figure 8:**
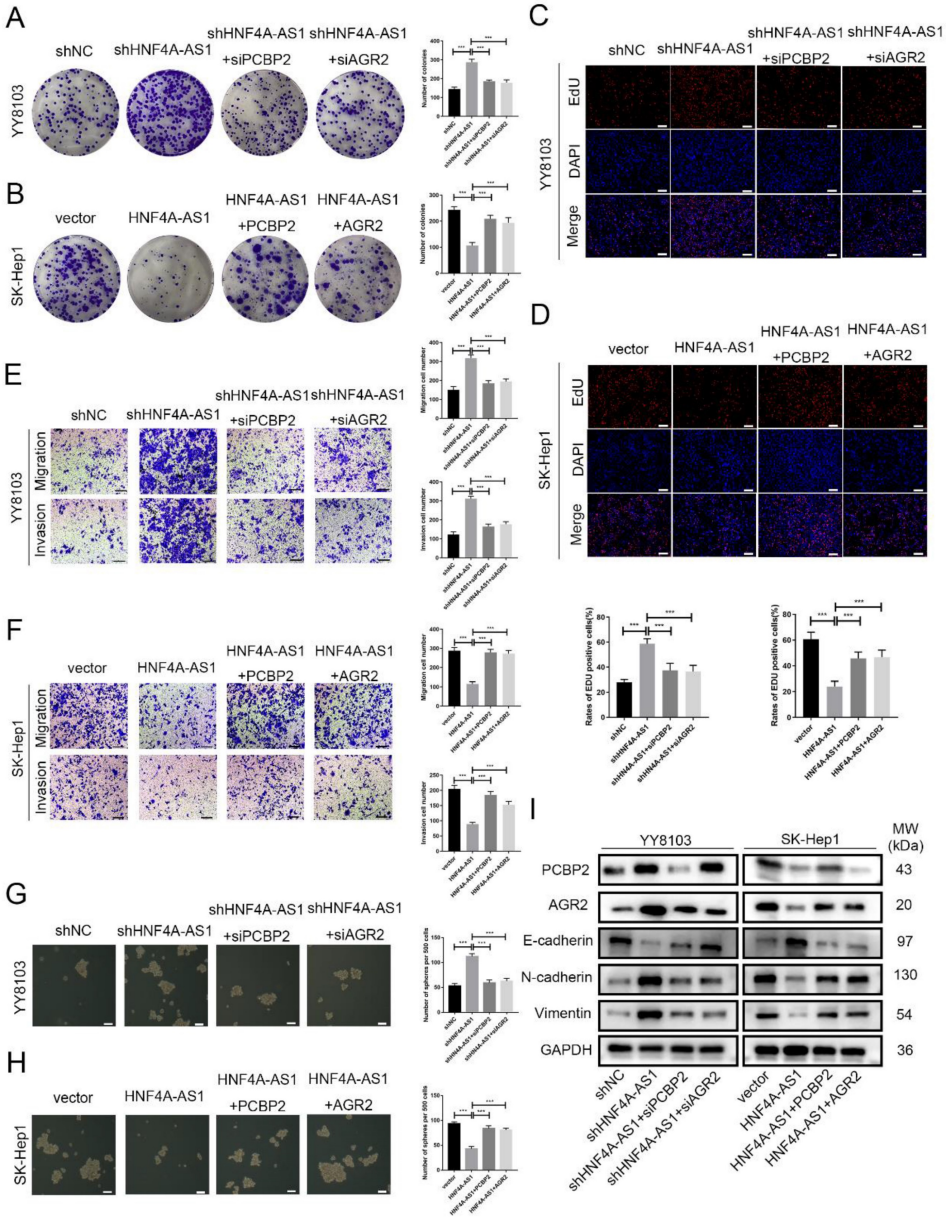
HNF4A-AS1 inhibits the progression of HCC through the PCBP2/AGR2 axis. A-D. Colony formation (A, B) and EdU assays (C, D) demonstrated that the knockdown of AGR2 or PCBP2 restored the proliferation of HNF4A-AS1-knockdown YY8103 cells, whereas the overexpression of AGR2 or PCBP2 enhanced the proliferation of SK-Hep1-HNF4A-AS1 cells. Scale bar, 50 μm. E-F. Transwell assays demonstrated that knockdown of AGR2 or PCBP2 restored the invasion and migration of YY8103 cells with HNF4A-AS1 knockdown (E). Additionally, overexpression of AGR2 or PCBP2 counteracted the effect of HNF4A-AS1 overexpression on the metastasis of SK-Hep1 cells (F). Scale bar, 100 μm. G-H. Sphere-forming assays showed that AGR2 or PCBP2 restored the impact of HNF4A-AS1 on the stemness of HCC cells. I. EMT-associated proteins were detected by western blot analysis, which demonstrated that AGR2 or PCBP2 restored the inhibiting impact of HNF4A-AS1 on EMT in HCC cells. Bar graphs represent mean ± SEM (n=3, and ***P < 0.001).

**Figure 9:**
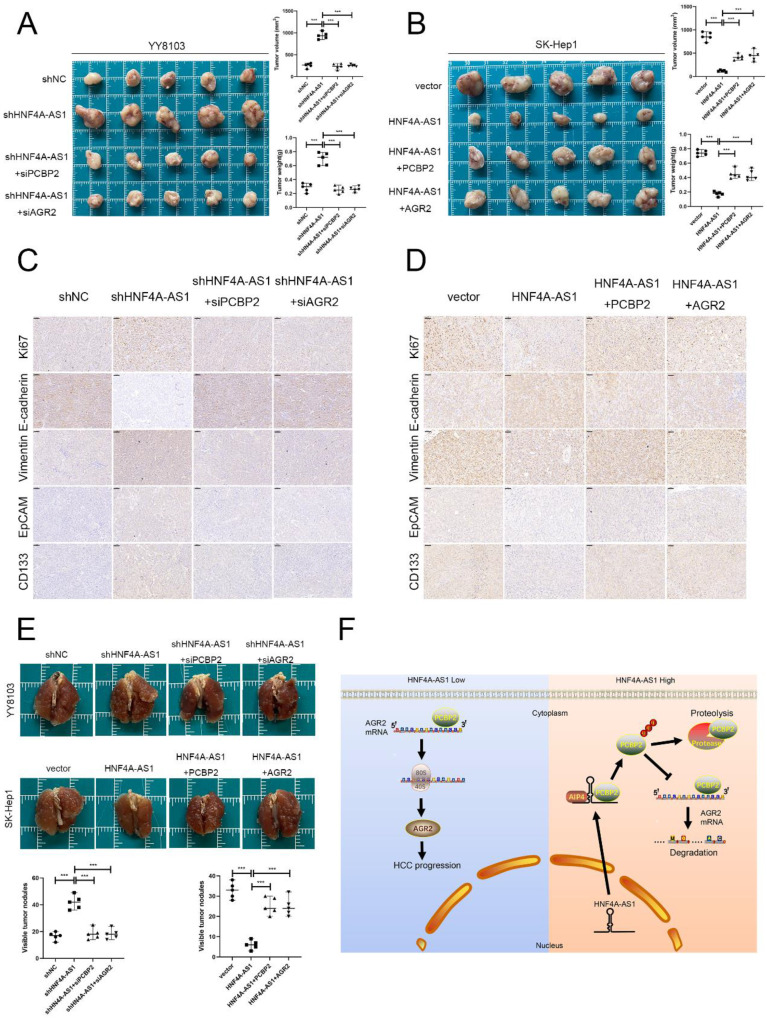
HNF4A-AS1 inhibits the progression of HCC through the PCBP2/AGR2 axis *in vivo*. A-B. A subcutaneous tumor model showed that the knockdown of AGR2 or PCBP2 restored the proliferation of HNF4A-AS1-knockdown YY8103 cells, whereas the overexpression of AGR2 or PCBP2 enhanced the proliferation of SK-Hep1-HNF4A-AS1 cells *in vivo*. The photograph of subcutaneous tumors (above), volume and weight of subcutaneous tumors (below). C-D. Immunohistochemistry of HE, Ki67, vimentin, E-cadherin, EpCAM, and CD133 in subcutaneous tumors. Scale bar, 50 μm. E. A lung metastasis model was generated to detect the metastasis of HCC cells. F. The schematic diagram illustrates the mechanism of action of HNF4A-AS1 in HCC. HNF4A-AS1 promotes the ubiquitination of PCBP2 protease to degrade and stabilize AGR2 mRNA, thereby facilitating the progression of HCC cells. Bar graphs represent mean ± SEM (n=3, and ***P < 0.001).

Rescue experiments were conducted to validate the functional role of the HNF4A-AS1/PCBP2/AGR2 axis in HCC. We initially knocked down PCBP2 or AGR2 in YY8103-shHNF4A-AS1 cells and overexpressed PCBP2 or AGR2 in SK-Hep1-HNF4A-AS1 cells. The results indicated that the knockdown of AGR2 or PCBP2 restored proliferation (Figure 8A, C), metastasis (Figure 8E), stemness (Figure 8G), and EMT signaling pathway (Figure 8I) activities in YY8103-shHNF4A-AS1 cells. Conversely, the overexpression of AGR2 or PCBP2 enhanced proliferation, metastasis, stemness, and EMT signaling pathway activities in SK-Hep1-HNF4A-AS1 cells (Figure 8B, D, F, H, I). We further examined the regulatory influence of AGR2 on the HNF4A-AS1/PCBP2 axis. Our findings indicated that the upregulation of AGR2 restored the functions associated with proliferation, metastasis, stemness, and the EMT signaling pathway in YY8103 cells with HNF4A-AS1 and PCBP2 knockdown. Conversely, downregulation of AGR2 reduced the aforementioned activities in SK-Hep1 cells with HNF4A-AS1 and PCBP2 overexpression (Figure S9A-E). Meanwhile, subcutaneous tumor model and lung metastasis model were used to validate the functional role of the HNF4A-AS1/PCBP2/AGR2 axis *in vivo*. The *in vivo* results were consistent with the *in vitro* results (Figure 9A-E, Figure 9F). In conclusion, HNF4A-AS1 regulates the malignancy of HCC through the PCBP2/AGR2 axis.

## Discussion

HCC is a malignancy that develops in individuals with various chronic liver diseases, including alcoholic fatty liver disease, viral hepatitis, and nonalcoholic fatty liver disease. The progression of HCC is typically preceded by chronic liver injury, which initiates a series of events including inflammation, activation of the extracellular matrix, regeneration, fibrosis, and ultimately cirrhosis^25^, ^26^. Current treatment options for HCC include hepatectomy, liver transplantation, and radiofrequency ablation for early-stage HCC. Transarterial embolization, radiotherapy, and chemotherapy are employed for advanced cases of HCC. However, despite these interventions, the prognosis for patients with HCC remains unsatisfactory due to high rates of recurrence and metastasis. Consequently, there is a pressing need to investigate the underlying molecular mechanisms driving the development of HCC in order to identify novel targets for clinical diagnosis and treatment.

HNF4A-AS1, the antisense strand of HNF4A, is highly expressed in liver tissue. It plays a regulatory role in the expression of CYP3A4 by influencing the accumulation of PXR in the CYP3A4 promoter and modifying histones^27^. This regulation leads to the inhibition of P450 expression in the liver, preventing ritonavir-induced hepatotoxicity^28^. In HCC, HNF4A-AS1 is transcriptionally activated by HNF4α and downregulated in HCC tissues^29^, ^30^, suggesting its potential as a diagnostic marker for HCC.

However, the specific regulatory effects of HNF4A-AS1 on the biological function of HCC and its underlying mechanism have not been thoroughly investigated. In our study, we observed a decrease in the levels of HNF4A-AS1 in HCC, which exhibited a positive correlation with the prognosis of HCC. Through cell and animal experiments, we have demonstrated that HNF4A-AS1 inhibits the migration, proliferation, and stemness of HCC cells. Additionally, it suppresses the EMT pathway. Notably, in a DEN-induced spontaneous HCC model, the overexpression of HNF4A-AS1 significantly reduced the incidence of HCC and prolonged overall survival in mice. These findings suggest that HNF4A-AS1 is involved in the initiation and progression of HCC and may serve as a potential therapeutic target for this disease. Interestingly, Song et al. found that sPEP1, encoded by HNF4A-AS1, promotes the binding of the amino and carboxyl terminals of eEF1A1 with SMAD4^31^. This interaction leads to reduced transactivation of SMAD4, thereby promoting the stem cell activity of neuroblastoma (NB) cells. Our analysis using coding potential assessment tools indicates that HNF4A-AS1 is a non-coding RNA. Additionally, proteins interacting with HNF4A-AS1 in BE(2)-C cells are involved in protein translation or ribosome assembly, while GO enrichment analysis of proteins interacting with HNF4A-AS1 in HCC cells did not reveal relevant functions. Therefore, we speculate that the low coding potential of HNF4A-AS1 in HCC may lead to differential regulation of stemness in NB and HCC cells. However, further exploration is needed to determine the possibility of HNF4A-AS1 encoding short peptides in HCC.

HNF4A-AS1 is an antisense RNA located between the HNF4α P1 and P2 promoters. HNF4α plays a critical role in liver development and function, functioning as a tumor suppressor gene in HCC^32^. The HNF4A gene is regulated by two distinct P1 and P2 promoters, with a significant decrease in P1-HNF4α expression observed in HCC cells^33^. Research has shown that HNF4α can activate its own promoter^34^. Guo et al. demonstrated that P1-HNF4α can stimulate the proximal promoter of HNF4A-AS1^29^. It is hypothesized that the decreased expression of HNF4A-AS1 in HCC may be regulated by HNF4α. However, further investigation is necessary to elucidate the precise underlying mechanism.

PCBP2 possesses three RNA-binding domains that exhibit a high affinity for binding C-rich polypyrimidine motifs^35^, ^36^. Previous studies have indicated that the PCBP2 protein is significantly upregulated in various types of cancer, such as hepatocellular carcinoma^37^, glioblastoma^38^, and pancreatic cancer^39^. PCBP2 plays a role in regulating the expression of downstream target genes by promoting their transcription^40^, maintaining the stability of their mRNA^16^, and facilitating their translation^17^. PCBP2 has been identified as a key regulator of tumor progression through its modulation by ubiquitination. Specifically, the interaction between PHGDH and PCBP2 has been shown to impede the ubiquitination-mediated degradation of PCBP2, thereby suppressing ferroptosis and facilitating malignant progression in bladder cancer^41^. Nevertheless, the mechanisms underlying the ubiquitination-mediated regulation of PCBP2 in HCC remain unclear. In this particular study, PCBP2 was identified as a binding protein of HNF4A-AS1 through pulldown and mass spectrometry analyses. It was further demonstrated that HNF4A-AS1 binds to the KH3 domain of PCBP2 through segment 3. HNF4A-AS1 facilitates the recruitment of the HECT domain-containing E3 ligase, AIP4, by PCBP2, thereby promoting the ubiquitination and subsequent degradation of PCBP2 by proteases.

In order to investigate the potential mechanism by which HNF4A-AS1 influences HCC cells, we conducted RNA sequencing on cells that were overexpressing HNF4A-AS1. Through the utilization of KEGG pathway and GO enrichment analysis, we have identified that HNF4A-AS1 plays a significant role in regulating multiple pathways that are closely linked to tumorigenesis. Furthermore, the gene distribution analysis, using GO enrichment analysis of the RNA-seq results, revealed that AGR2 is implicated in the majority of pathways related to tumors. AGR2 is a human homolog of Xenopus anterior gradient-2 (XAG-2) and possesses a secreted signal peptide, which allows it to be secreted into the extracellular compartment^42^. AGR2 has been closely linked to the initiation of cancer, tumor progression, and metastasis^43^, ^44^. Previous studies have reported that AGR2 enhances the invasiveness of tumor cells in colorectal and breast malignancies^45^, ^46^. In HCC, studies have shown that AGR2 is involved in sorafenib resistance and plays a role in microRNA-mediated cell proliferation and metastasis^47^, ^48^. Our study demonstrated that AGR2 promotes the proliferation, invasion, and stemness of HCC cells. Meanwhile, our findings revealed that PCBP2 binds to the 3' UTR of AGR2 mRNA and enhances its stability, while HNF4A-AS1 inhibits the stability of AGR2 mRNA by promoting the degradation of PCBP2. Functional experiments provided evidence that HNF4A-AS1 regulates the function of HCC through the PCBP2/AGR2 axis.

## Conclusions

In summary, we found that HNF4A-AS1 is downregulated in HCC and is an independent risk factor for the prognosis of HCC. HNF4A-AS1 was found to inhibit the stability of AGR2 mRNA by promoting the proteasomal degradation of PCBP2, thereby playing a role in suppressing the metastasis, proliferation, and stemness of HCC cells (Figure 9F). Consequently, this study presents a new potential target for the diagnosis and treatment of HCC.

## Supplementary Material

Supplementary figures and tables.

## Figures and Tables

**Table 1:** Correlation between HNF4A-AS1 expression and clinicopathological features

Clinicopathological features	All cases		HNF4A-AS1		*p* value
			High expression	Low expression	
Age (years)	>60	69	39	30	
	≤60	31	11	20	0.0517
Gender	Female Male	41 59	17 33	24 26	0.0619
HBV	Negative	18	8	10	
	Positive	82	42	40	0.6027
Tumor multiplicity	Single	62	41	21	
	Multiple	38	19	29	0.0056**
Tumor size (cm)	≤5	54	32	22	
	>5	46	18	28	0.0480*
α-fetoprotein (ng/ml)	≤200	33	15	18	
	>200	67	35	32	0.8217
Edmondson stage	I-II	65	39	26	
	III-IV	35	11	24	0.0064**
TNM stage	I	58	34	24	
	II-III	42	16	26	0.0428*
Microvascular invasion	Yes	33	11	22	
	No	67	39	28	0.0193*

*p < 0.05, **P < 0.01.

**Table 2:** Univariate and multivariate analysis of factors associated with overall survival of 100 HCC patients

Clinicopathologic Parameters	Univariable analysis			Multivariable analysis			
	HR	95%CI	P value		HR	95%CI	P value
Age (>50 years vs ≤ 50 years)	1.1	0.56-2.1	0.8225				
Gender (female vs male)	0.84	0.46-1.5	0.5634				
HBV infection (positive vs negative)	0.87	0.4-1.9	0.7343				
TNM stage (II/III vs I)	2.3	1.3-4.3	**0.0059**				
Microvascular invasion (yes vs no)	2.6	1.4-4.8	**0.0028**		1.800	0.933-3.471	**0.0450**
Tumor multiplicity (multiple vs simple)	0.38	0.21-0.7	**0.0021**		1.477	0.728-2.999	0.2831
α-fetoprotein (≥20 ng/ml vs < 20 ng/ml)	0.34	0.079-4.3	0.7210				
Edmonson stage (III/IV vs I/II)	2.3	1.2-4.3	**0.0079**				
Tumor size (≥5 cm vs < 5 cm)	2.1	1.1-3.8	**0.0173**		1.508	0.786-2.892	0.2163
HNF4A-AS1 expression (high vs low)	0.35	0.18-0.66	**0.0012**		0.502	0.248-1.013	**0.0421**
							

*p < 0.05, **p < 0.01, ***p < 0.001. HR, hazard ratio; CI, confidence interval.

## Abbreviations

HCC: Hepatocellular Carcinoma
HNF4A-AS1: Hepatocyte nuclear factor 4 alpha antisense RNA 1
PCBP2: Poly(rC)-binding Protein 2
AGR2: Anterior Gradient 2
TMA: Tissue microarray
OS: Overall survival
DFS: Disease-free survival
IHC: Immunochemistry staining
FISH: Fluorescence *in situ* hybridization
CCK-8: Cell counting Kit-8
EDU: 5-Ethynyl-20-deoxyuridine
siRNA: small interfering RNA
shRNA: short hairpin RNA
Co-IP: Co-immunoprecipitation
IF: Immunofluorescence
EMT: Epithelial Mesenchymal Transformation
TCGA: The Cancer Genome Atlas
KEGG: Kyoto Encyclopedia of Genes and Genomes
GO: Gene Ontology
