# Supplementary material for: HNF4A-AS1 inhibits the progression of hepatocellular carcinoma by promoting the ubiquitin-modulated degradation of PCBP2 and suppressing the stability of ARG2 mRNA
Source: Int J Biol Sci. 2024 Sep 23;20(13):5087–108. doi: 10.7150/ijbs.95276 (PMC11488582; doi:10.7150/ijbs.95276)
Supplement: Supplementary file 1 — Supplementary figures and tables. [file ijbsv20p5087s1.zip › Supplemental Figures and Tables.pdf]

**HNF4A-AS1 inhibits the progression of hepatocellular carcinoma by promoting the ubiquitin-modulated degradation of PCBP2 and suppressing the stability of ARG2 mRNA**

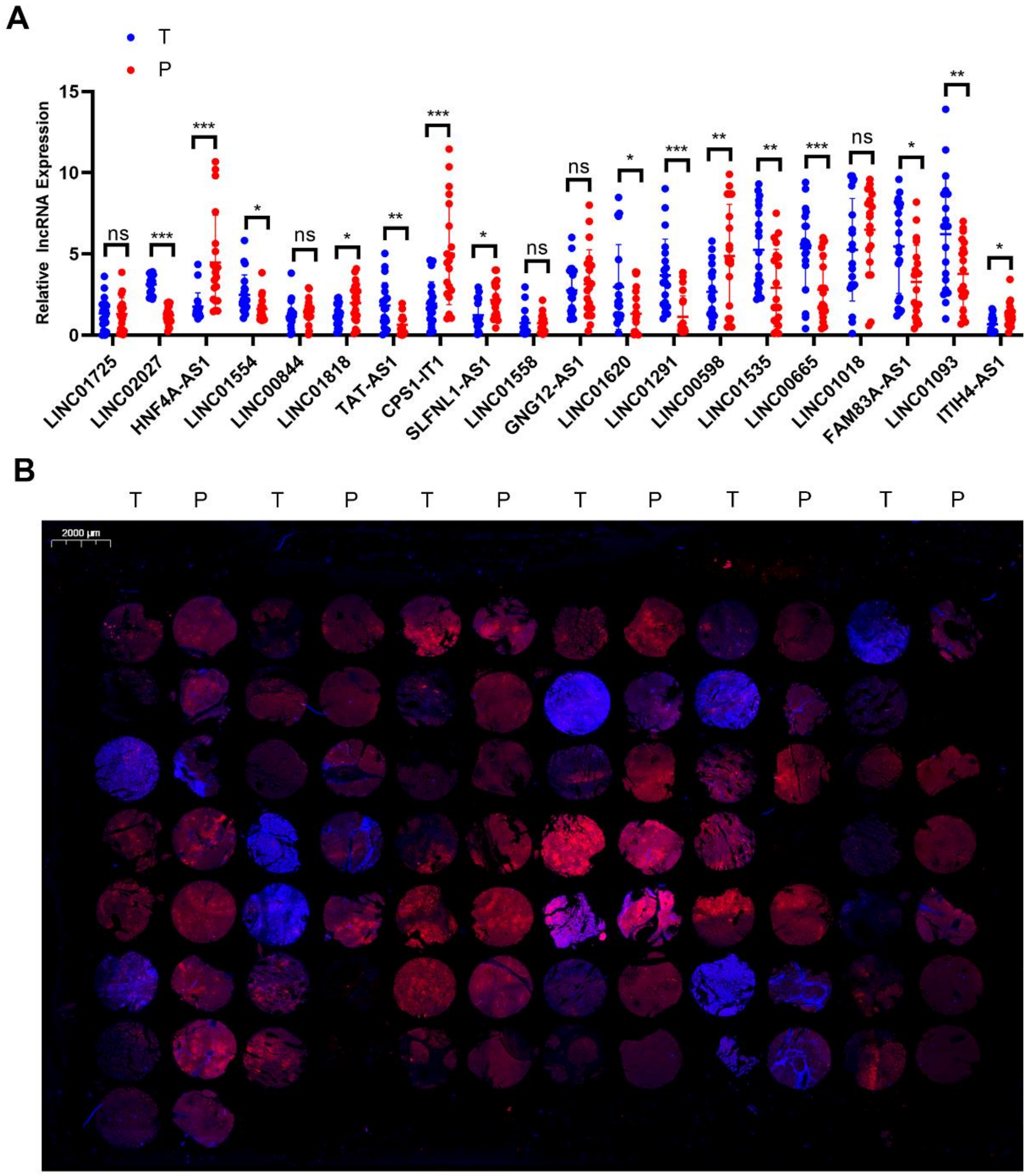


**Figure S1** The expression of HNF4A-AS1 is decreased in HCC. A. The expression of 20 candidate lncRNAs in 20 pairs of HCC and paracarcinoma tissues was detected by qRT-PCR. B. HNF4A-AS1 expression in 43 pairs of HCC tissues or paracarcinoma tissues was detected by qRT-PCR (T: HCC tissues, P: paracarcinoma tissues). Scale bar, 2000 μm.

Bar graphs represent mean ± SEM (n=3, *P < 0.05, **P < 0.01, and ***P < 0.001).

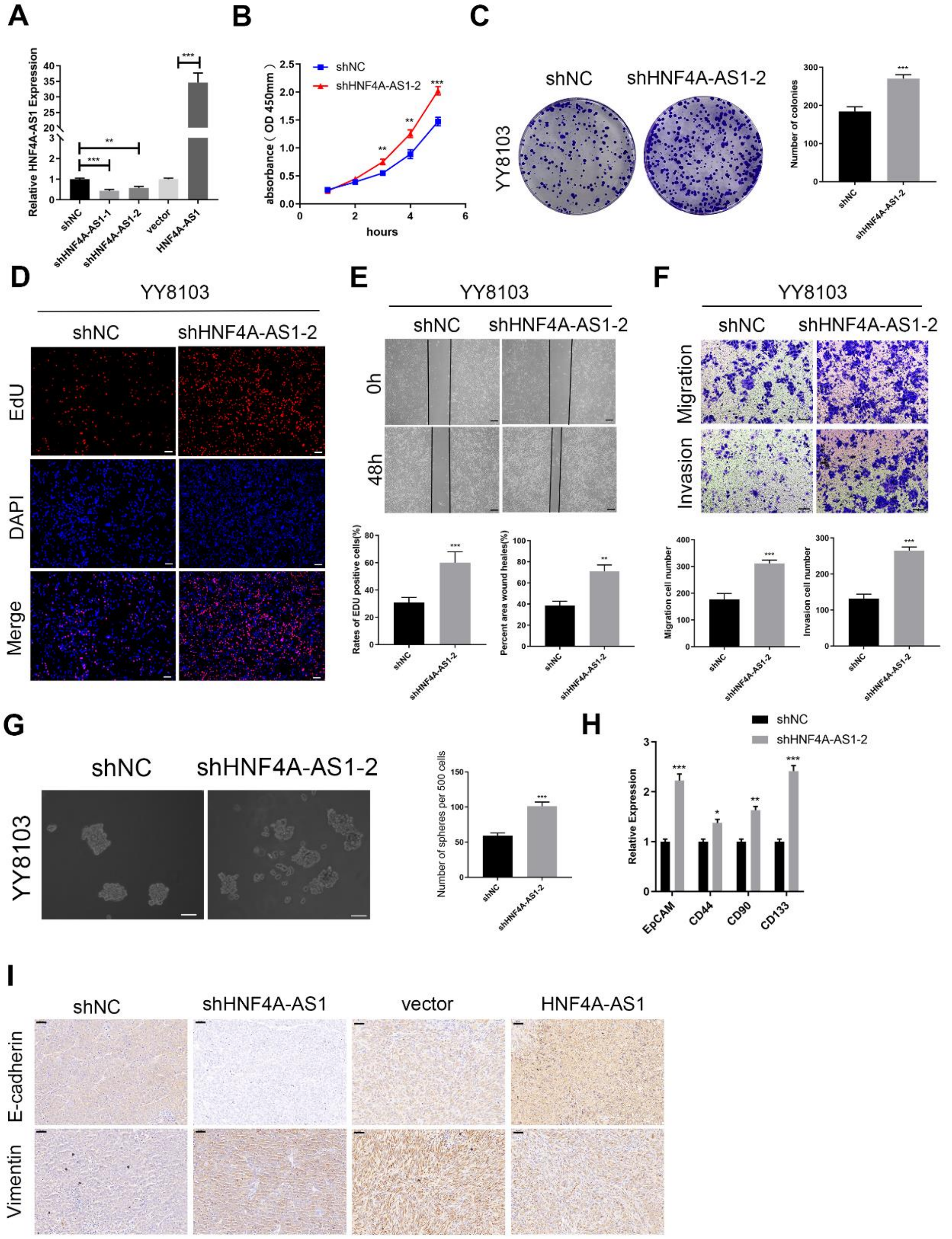


**Figure S2** HNF4A-AS1 suppresses proliferation, metastasis and stemness of HCC cells. A. The qRT-PCR was used to confirm the effectiveness of knocking down and overexpressing HNF4A-AS1. B. The effect of HNF4A-AS1 knockdown on the proliferation of HCC cells was assessed using a CCK8 assay. C-D. A colony formation assay (C) and

EdU assay (D) presented that HNF4A-AS1 knockdown promoted the proliferation of YY8103 cells. Scale bar, 50 μm. E. A wound-healing assay was used to determine the role of HNF4A-AS1 knockdown in the motility of HCC cells. Scale bar, 500 μm. F. Transwell assays showed that knockdown of HNF4A-AS1 promoted cell invasion and migration in YY8103 cells. Scale bar, 200 μm. G. Sphere-forming assays showed that spheroid formation was enhanced in HNF4A-AS1-deficient YY8103 cells. Scale bar, 100 μm. H. The expression levels of CSC-related biomarkers (EpCAM, CD133, CD44, and CD90) in spheroids were detected by qRT-PCR. I. Immunohistochemistry of E-cadherin and Vimentin in subcutaneous tumors. Scale bar, 50 μm. Bar graphs represent mean ± SEM (n=3, *P < 0.05, **P < 0.01, and ***P < 0.001).

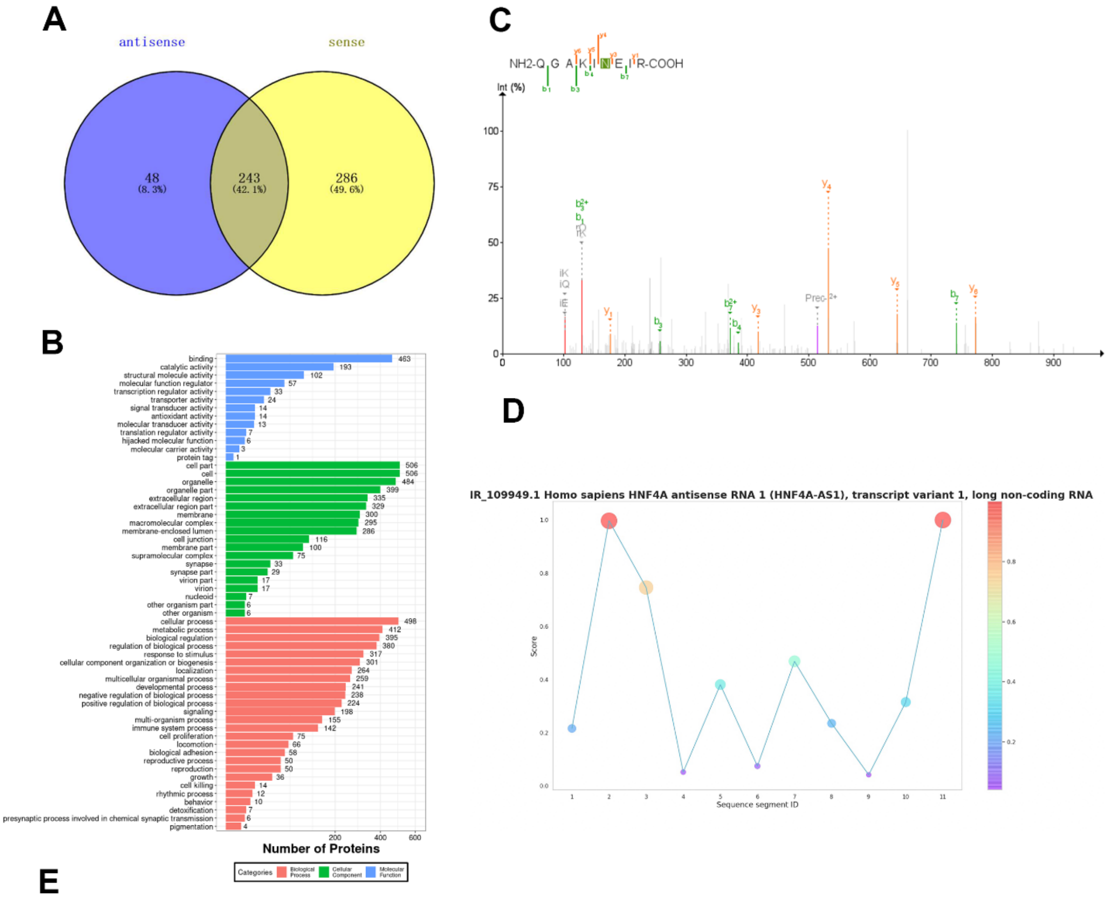


E

segment 1：

CTAACTATCTCACAATATAGGTCCCCAACCACTGACCAAACTCCAGTCCAGGCAGCCAC-
CAGCTGGCCTGGTCTTGCTGCTTCCTTTAGCGGCTTCCAAGGTCCAGGGACAGGGGGTCTGG
GCCACCAAGAGGCTCTGCTAGGCTGTCCCTGCAGCCACAGCCACCCCACTGGACCCCTGCCT
CCCATCTGAGAGGACAGCCCTCTTCCTGGAGCTGGG

segment 2：

ATCTGACACTTGCTGATACCAACGGCAGATACCAGGTCAAGGATTCGGTGACTTGAAGA-
CAGGCCTCCTCTGTTGCTTTGAGTCAATTTCTAGTACCTGGGCCTAAATCTTGACTGCACACCG
GTCATATTATTGAGGTAACGAGATGTGAGCAGAAACAACCTGTGTCACACCTGGGCAGAAGCT
TTAAAGGCCAGTGCTGCCCTTGTTGGGGAAGCAAGTATAGATATGAATCGTTCCACAACGCGG
AGCGCCCCCTGCCGACCCATGC

segment 3：

TGGGCGTGTACTATAGGTATAAGAATAGTCTTTGGAGCATCATCTGAGATAGTGAGC-
CAAGATCGTGCAACTGCACTCCAGCCTGAGTAACAGAGTGAGACTCCATCTCAAAACAAACAA
ATAAACAAAACAGAATAAATATACTCAGGATATTTACAAAAAAAAAAAAAAAAAAAAAAAAA

**Figure S3** HNF4A-AS1 physically interacts with PCBP2. A. A Venn diagram illustrates the binding proteins of HNF4A-AS1 sense and antisense. B. GO enrichment analysis was conducted on the binding proteins of HNF4A-AS1. C. Mass spectrometry (MS) analysis

was employed to determine the binding of PCBP2 to HNF4A-AS1. D. The score of the PCBP2 binding site of HNF4A-AS1, date from csbio. (http://www.csbio.sjtu.edu.cn) E. The deletion mutant sequence of deletion mutants.

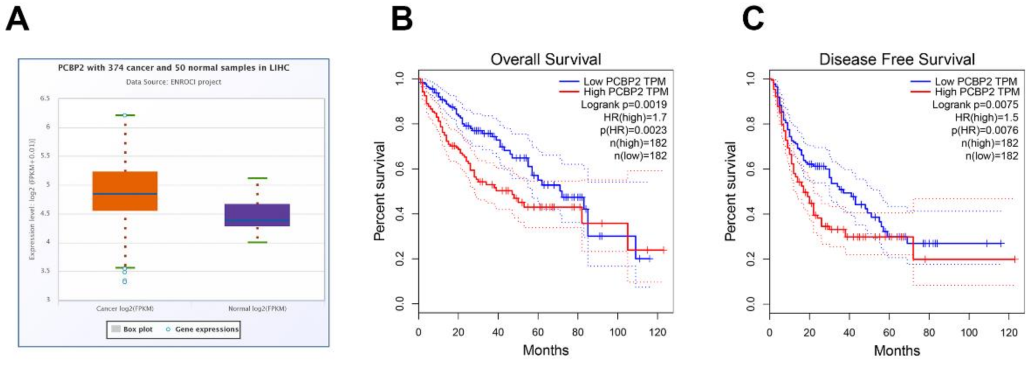


**Figure S4** PCBP2 was associated with the prognosis of HCC patients. A. PCBP2 was upregulated in HCC tissues. Data from Starbase. B-C. Overall survival and disease-free survival in HCC patients with high or low expression of PCBP2. Data from GEPIA.

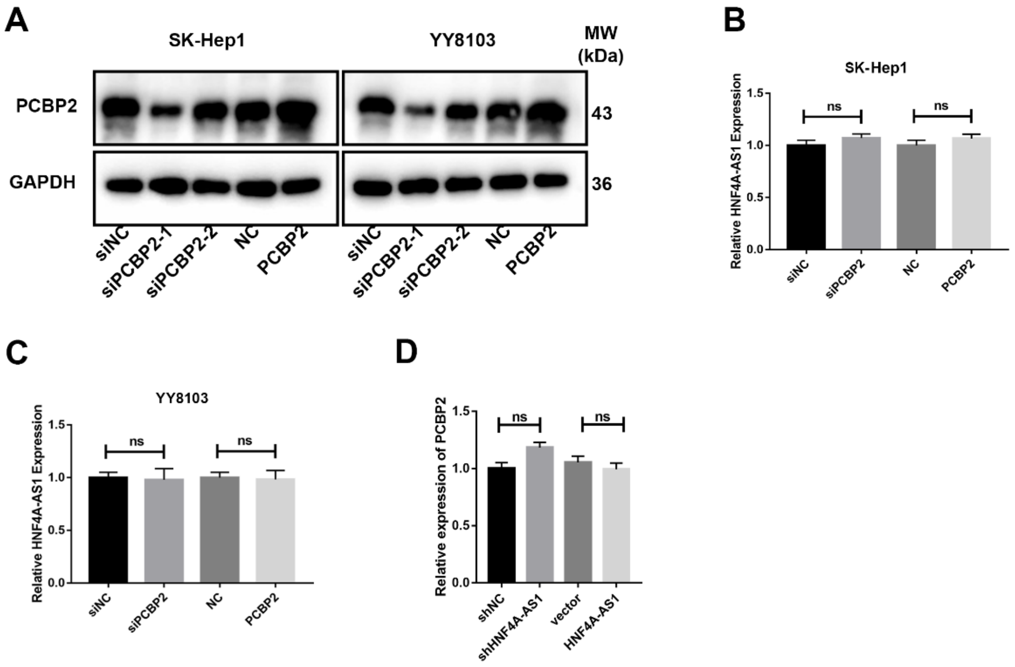


**Figure S5** PCBP2 had no regulatory effect on HNF4A-AS1. A. The knockdown and overexpression efficiency of PCBP2 were detected through western blot analysis. B-C. The expression of HNF4A-AS1 was detected by qRT-PCR after PCBP2 knockdown or

overexpression in SK-Hep1 and YY8103 cells. D. qRT-PCR showed that knockdown and levels of HNF4A-AS1 did not affect PCBP2 mRNA expression. Bar graphs represent mean ± SEM (n=3, *P < 0.05, **P < 0.01, and ***P < 0.001).

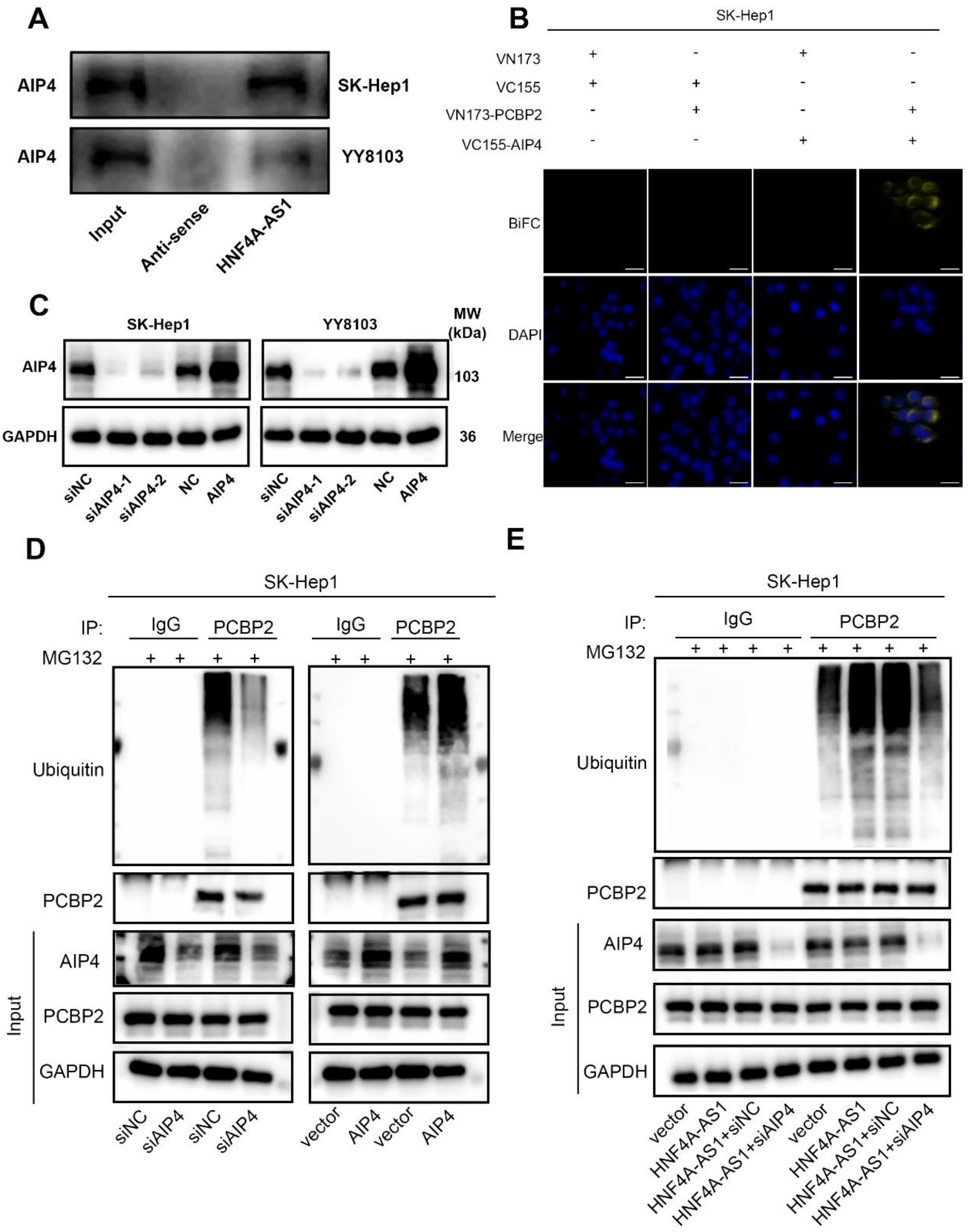


**Figure S6** AIP4 was a direct binding partner of HNF4A-AS1. A. Pull-down assays showed that AIP4 was a direct binding partner of HNF4A-AS1. B. The BiFC assay revealed that

AIP4 and PCBP2 were bound in the SK-Hep1 cells. Scale bar, 50 μm. C. The knockdown and overexpression efficiency of AIP4 was detected through western blot analysis. D. AIP4 knockdown rescued the promoting effect of overexpressed HNF4A-AS1 on PCBP2 ubiquitination. E. Western blot analysis showed that AIP4 promoted PCBP2 ubiquitination in SK-Hep1 cells.

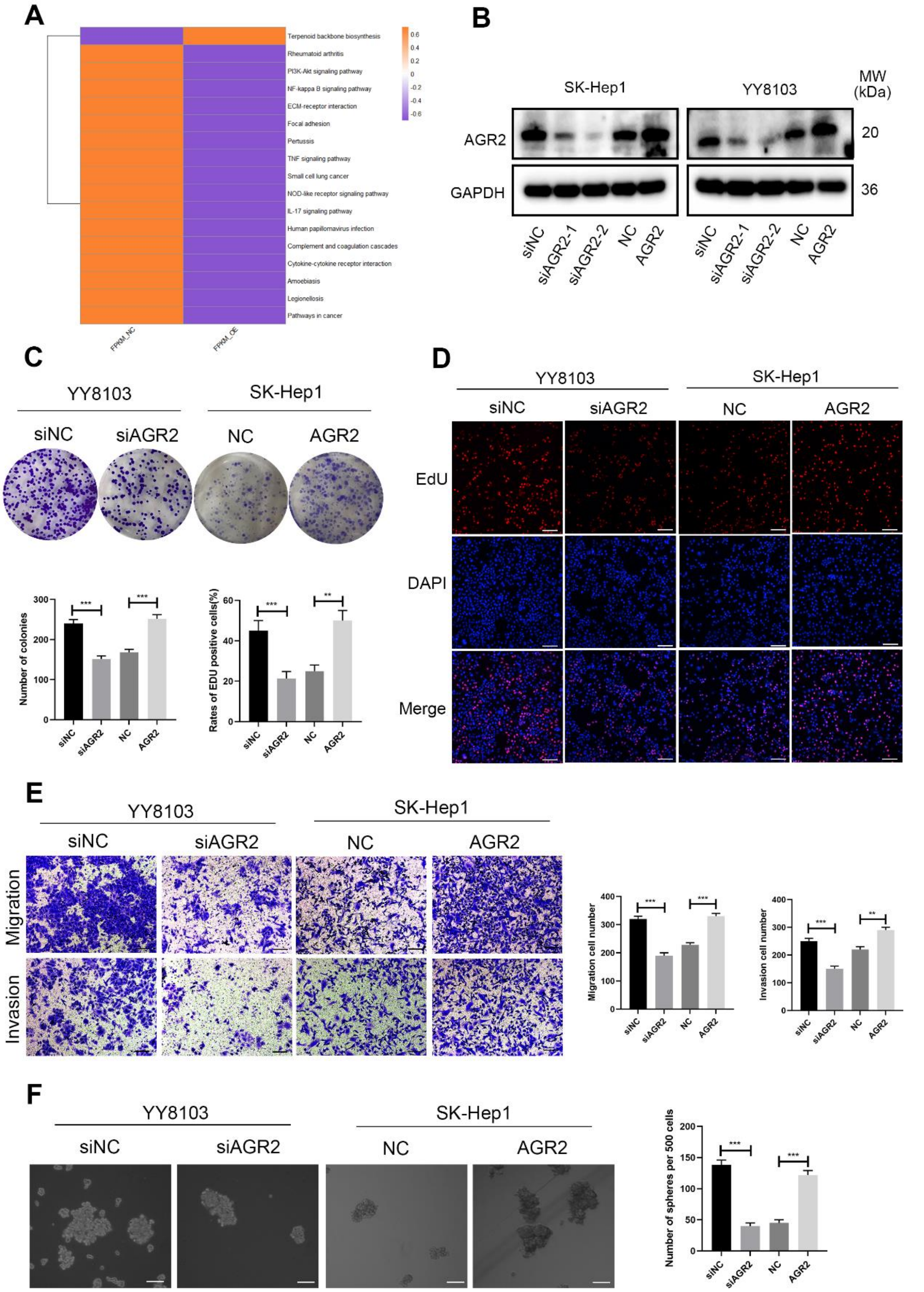


**Figure S7** AGR2 promotes proliferation, metastasis, and stemness of HCC cells. A. The heatmap shows the results of KEGG pathway analysis based on RNA-seq data from three pairs of HNF4A-AS1-overexpressing SK-Hep1 cells and negative control cells (NC:

negative control, OE: HNF4A-AS1-overexpressing). B. The knockdown and overexpression efficiency of AGR2 were detected through western blot analysis. C-D. A colony formation assay (C) and EdU assay (D) presented that the overexpression of AGR2 promoted the proliferation of SK-Hep1 cells, while AGR2 knockdown suppressed YY8103 cell proliferation. Scale bar, 50 μm. E. Transwell assays showed that knockdown of AGR2 inhibited cell invasion and migration in YY8103 cells, while AGR2 overexpression suppressed the migration and invasion capacities of SK-Hep1 cells. Scale bar, 200 μm. F. Sphere-forming assays showed that spheroid formation was attenuated in AGR2-deficient YY8103 cells, but enhanced in AGR2-overexpressing SK-Hep1 cells. Scale bar, 100 μm. Bar graphs represent mean ± SEM (n=3, *$P < 0.05$, **$P < 0.01$, and ***$P < 0.001$).

A

3’UTR Exons：

AGAAAAAAAATCTCCAAGCCCTTCTGTCTGTCAGGCCTTGAGACTTGAAACCAGAAGAAGT-
GTGAGAAGACTGGCTAGTGTGGAAGCATAGTGAACACACTGATTAGGTTATGGTTTAATGTTACAACA
ACTATTTTTTAAGAAAAACAAGTTTTAGAAATTTGGTTTCAAGTGTACATGTGTGAAAACAATATTGTAT
ACTACCATAGTGAGCCATGATTTTCTAAAAAAAAAAAATAAATGTTTTGGGGGTGTTCTGTTTTCTCCAA
CTTGGTCTTTCACAGTGGTTCGTTTACCAAATAGGATTAAACACACACAAAATGCTCAAGGAAGGGAC
AAGACAAAACCAAAACTAGTTCAAATGATGAAGACCAAAGACCAAGTTATCATCTCACCACACCACAG
GTTCTCACTAGATGACTGTAAGTAGACACGAGCTTAATCAACAGAAGTATCAAGCCATGTGCTTTAGC
ATAAAAGAATATTTAGAAAAACATCCCAAGAAAATCACATCACTACCTAGAGTCAACTCTGGCCAGGAA
CTCTAAGGTACACACTTTCATTTAGTAATTAAATTTTAGTCAGATTTTGCCCAACCTAATGCTCTCAGG
GAAAGCCTCTGGCAAGTAGCTTTCTCCTTCAGAGGTCTAATTTAGTAGAAAGGTCATCCAAAGAACAT
CTGCACTCCTGAACACACCCTGAAGAAATCCTGGGAATTGACCTTGTAATCGATTTGTCTGTCAAGGT
CCTAAAGTACTGGAGTGAAATAAATTCAGCCAACATGTGACTAATTGGAAGAAGAGCAAAGGGTGGT
GACGTGTTGATGAGGCAGATGGAGATCAGAGGTTACTAGGGTTTAGGAAACGTGAAAGGCTGTGGCA
TCAGGGTAGGGGAGCATTCTGCCTAACAGAAATTAGAATTGTGTGTTAATGTCTTCACTCTATACTTAA
TATATGGAATTCCTCTACTGCCCAGCCCCTCCTGATTTCTTTGGCCCCTGGACTATGGT-
GCTGTATATAATGCTTTGCAGTATCTGTTGCTTGTCTTGATTAACTTTTTTGGATAAAACCTTTTTTGAA
CAGA

5’UTR Exons：

ACTCAGAAGCTTGGACCGCATCCTAGCCGCCGACTCACACAAGGCAGGTGGGTGAG-
GAAATCCAGAGTTGCC

CDS Exons:

ATGGAGAAAATTCCAGTGTCAGCATTCTTGCTCCTTGTGGCCCTCTCCTACACTCTGGCCA-
GAGATACCACAGTCAAACCTGGAGCCAAAAAGGACACAAAGGACTCTCGACCCAAACTGCCCCAGA
CCCTCTCCAGAGGTTGGGGTGACCAACTCATCTGGACTCAGACATATGAAGAAGCTCTATATAAATC
CAAGACAAGCAACAAACCCTTGATGATTATTCATCACTTGGATGAGTGCCCACACAGTCAAGCTTTAA
AGAAAGTGTTTGCTGAAAATAAAGAAATCCAGAAATTGGCAGAGCAGTTTGTCCTCCTCAATCTGGTT
TATGAAACAACTGACAAACACCTTTCTCCTGATGGCCAGTATGTCCCCAGGATTATGTTTGTTGACCC
ATCTCTGACAGTTAGAGCCGATATCACTGGAAGATATTCAAATCGTCTCTATGCTTACGAACCTGCAG
ATACAGCTCTGTTGCTTGACAACATGAAGAAAGCTCTCAAGTTGCTGAAGACTGAATTGTAA

**Figure S8** A. The full-length, 3’UTR, 5’UTR, and CDS sequences of AGR2 mRNA (data from UCSC).

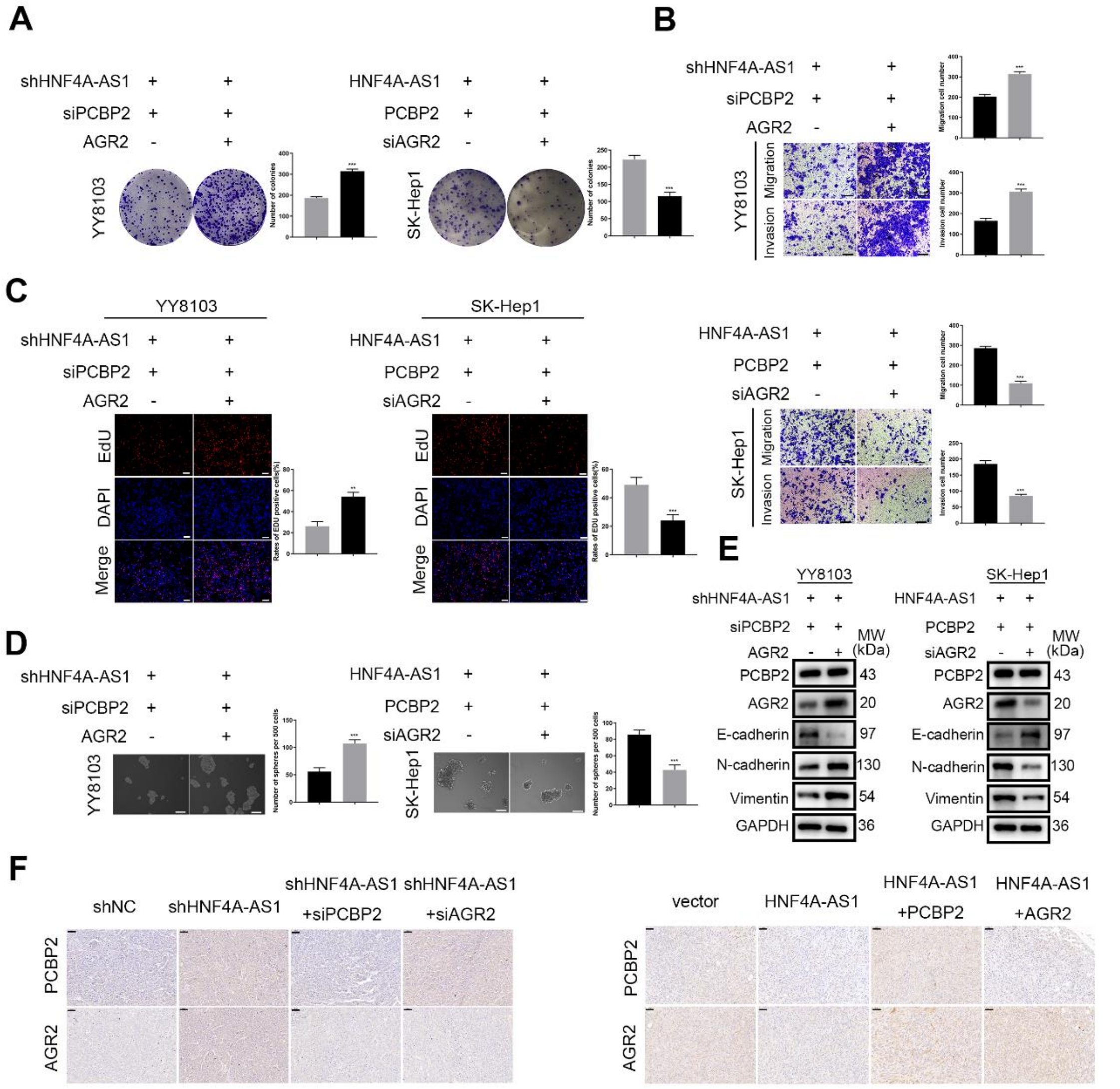


**Figure. S9** HNF4A-AS1/PCBP2 regulates the progression of HCC through AGR2. A-B. Colony formation (A) and EdU assays (B) showed that the overexpression of AGR2 restored the proliferation of HNF4A-AS1-knockdown and PCBP2-knockdown YY8103 cells, while the knockdown of AGR2 reduced the proliferation of SK-Hep1-HNF4A-AS1+PCBP2 cells. Scale bar, 50 μm. C. Transwell assays demonstrated that overexpression of AGR2 counteracted the effect of HNF4A-AS1 and PCBP2 overexpression on the metastasis of YY8103 cells. Additionally, knockdown of AGR2 restored the invasion and migration of SK-Hep1 cells with HNF4A-AS1 and PCBP2 knockdown. Scale bar, 100 μm. D. Sphere-forming assays showed that AGR2 restored the impact of HNF4A-AS1/PCBP2 on the

stemness of HCC cells. E. EMT-associated proteins were detected by western bolt analysis, which demonstrated that AGR2 restored the inhibiting impact of HNF4A-AS1/PCBP2 on EMT in HCC cells. F. Immunohistochemistry of PCBP2 and AGR2 in subcutaneous tumors. Scale bar, 50 μm. Bar graphs represent mean ±SEM (n=3, *P < 0.05, **P < 0.01 and ***P < 0.001)

**Supplemental table 1** Sequence for shRNA and siRNA

| | |
|---|---|
| shHNF4A-AS1 | CCAACCACTGACCAAACTCC |
| | GACTCGTTCCACAACGCGGA |
| si-PCBP2 | CCATGATCCATCTGTGTAGTT |
| | CCCACTAATGCCATCTTCAAA |
| si-AGR2 | TTCTGAGTTAGCAACAAGTAA |
| | AAGCTCTATATAAATCCAAGA |
| si-AIP4 | AAGTGCTTCTCAGAATGATGA |
| | AACCACAACACACGAATTACA |

**Supplemental table 2** Primers used for quantitative RT-PCR

| Target | Direction | Sequence |
|---|---|---|
| HNF4A-AS1 | Forward | 5’- TCGGTGACTTGAAGACAGGC -3’ |
| | Reverse | 5’- CCAGGTGTGACACAGGTTGT -3’ |
| PCBP2 | Forward | 5’- CTTTGGCTGGACCCACTAATG-3’ |
| | Reverse | 5’- CCCTGTACTCTCTCGTATTTCCT-3’ |
| AGR2 | Forward | 5’- AGGACTCTCGGCCCAAACTA-3’ |
| | Reverse | 5’- GCTTGACTGTGTGGGCATTC-3’ |
| EpCAM | Forward | 5′-AATCGTCAATGCCAGTGTACTT-3′ |
| | Reverse | 5′-TCTCATCGCAGTCAGGATCATAA-3′ |
| CD44 | Forward | 5’-TCCAACACCTCCCAGTATGACA-3’ |
| | Reverse | 5’-GGCAGGTCTGTGACTGATGTACA-3 |
| CD133 | Forward | 5'-ACTCGG CTCCCTGTTGCTGCT-3' |
| | Reverse | 5'-GAGCCTGATATC CTGACCATTG-3' |
| CD90 | Forward | 5’-TCACCCATCCAGTACGAGTTC-3’ |
| | Reverse | 5’-GGAGCGGTATGTGTGCTCAG-3’ |

| | | |
|---|---|---|
| GAPDH | Forward | 5′-GGGGCAGTTATTGCACTTGTC−3′ |
| | Reverse | 5′-AGGGGCCATCCACAGTCTTC-3′ |

**Supplemental table 3** Antibodies used in this study.

| Antibody | Company | Catalog Number |
|---|---|---|
| PCBP2 | Proteintech | 15070-1-AP |
| AIP4 | Proteintech | 20920-1-AP |
| AGR2 | Proteintech | 12275-1-AP |
| E-Cadherin | Proteintech | 20874-1-AP |
| N-Cadherin | Proteintech | 66219-1-Ig |
| Vimentin | Proteintech | 10366-1-AP |
| Flag | Proteintech | 80010-1-RR |
| HA | Proteintech | 51064-2-AP |
| His | Proteintech | 66005-1-Ig |
| Ubiquitin | Cell Signaling Technology | 3936 |

**Supplemental Table 5**. CPAT prediction of HNF4A-AS1

| Sequence Name | RNA size | ORF size | Ficket Score | Hexamer Score | Coding Probability | Coding Label |
|---|---|---|---|---|---|---|
| NR_109949.1 | 672 | 90 | 0.9976 | 0.128086415 | 0.039751237 | no |
